# The Relationship between Host Lifespan and Pathogen Reservoir Potential: An Analysis in the System *Arabidopsis thaliana-Cucumber mosaic virus*

**DOI:** 10.1371/journal.ppat.1004492

**Published:** 2014-11-06

**Authors:** Jean Michel Hily, Adrián García, Arancha Moreno, María Plaza, Mark D. Wilkinson, Alberto Fereres, Aurora Fraile, Fernando García-Arenal

**Affiliations:** 1 Centro de Biotecnología y Genómica de Plantas (UPM-INIA), and E.T.S.I. Agrónomos, Campus de Montegancedo, Universidad Politécnica de Madrid, Madrid, Spain; 2 Centro de Biotecnología y Genómica de Plantas (UPM-INIA), Campus de Montegancedo, Universidad Politécnica de Madrid, Madrid, Spain; 3 Instituto de Ciencias Agrarias, Consejo Superior de Investigaciones Científicas (ICA-CSIC), Madrid, Spain; Oklahoma State University, United States of America

## Abstract

Identification of the determinants of pathogen reservoir potential is central to understand disease emergence. It has been proposed that host lifespan is one such determinant: short-lived hosts will invest less in costly defenses against pathogens, so that they will be more susceptible to infection, more competent as sources of infection and/or will sustain larger vector populations, thus being effective reservoirs for the infection of long-lived hosts. This hypothesis is sustained by analyses of different hosts of multihost pathogens, but not of different genotypes of the same host species. Here we examined this hypothesis by comparing two genotypes of the plant *Arabidopsis thaliana* that differ largely both in life-span and in tolerance to its natural pathogen *Cucumber mosaic virus* (CMV). Experiments with the aphid vector *Myzus persicae* showed that both genotypes were similarly competent as sources for virus transmission, but the short-lived genotype was more susceptible to infection and was able to sustain larger vector populations. To explore how differences in defense against CMV and its vector relate to reservoir potential, we developed a model that was run for a set of experimentally-determined parameters, and for a realistic range of host plant and vector population densities. Model simulations showed that the less efficient defenses of the short-lived genotype resulted in higher reservoir potential, which in heterogeneous host populations may be balanced by the longer infectious period of the long-lived genotype. This balance was modulated by the demography of both host and vector populations, and by the genetic composition of the host population. Thus, within-species genetic diversity for lifespan and defenses against pathogens will result in polymorphisms for pathogen reservoir potential, which will condition within-population infection dynamics. These results are relevant for a better understanding of host-pathogen co-evolution, and of the dynamics of pathogen emergence.

## Author Summary

Understanding pathogen emergence is a major goal of pathology, because of the high impact of emerging diseases. Pathogens emerge onto a new host from a reservoir, hence the relevance of identifying the determinants of host's reservoir potential. Host lifespan is considered as one such determinant: short-lived hosts will invest less in defenses, being more susceptible to infection, more competent as infection sources and/or will sustain larger vector populations, and thus, are effective reservoirs for long-lived host infection. Evidence for this hypothesis derives from analyses of different hosts of multihost pathogens, and here we examine whether it holds at the within-species level by comparing two genotypes of the plant *Arabidopsis thaliana* that differ in life-span and in tolerance to its natural pathogen *Cucumber mosaic virus*. Experiments showed that defenses to the virus and its aphid vector were less efficient in the short-lived genotype that, according to model simulations, was an effective reservoir under a large range of conditions. Reservoir potential, though, was modulated by the demography of host and vector and by the genetic composition of the host population. Thus, within-species genetic diversity for lifespan and pathogen defense will result in differences in reservoir potential, which will condition infection dynamics and host-pathogen co-evolution.

## Introduction

Understanding the complex interplay of factors resulting in pathogen emergence has been a major goal of evolutionary ecology for the last twenty years, as emerging infectious diseases often have a high impact in human and animal health, agriculture and conservation [1]–[5]. Generally, emergent pathogens are multi-host pathogens that spill-over onto a new host population from one or more epidemiologically connected populations in which the pathogen can be permanently maintained, that is, from a reservoir host *sensu* Haydon et al. [2]. Hence, identifying the causes that determine the reservoir potential of a host, i.e., its ability to sustain pathogen populations for transmission to the target host, is central for understanding emergence and, more generally, infection dynamics. Emergent pathogens are often vector-transmitted [1], [3], [4], so that parameters associated with the triple interaction host-pathogen-vector should be taken into consideration for predicting host reservoir potential. For vector-transmitted pathogens, three epidemiological parameters have been underscored as modulating host reservoir potential: i) the probability that a vector acquires the pathogen when feeding on an infected host (host competence), ii) the probability that a host is infected by a feeding vector that carries the pathogen (host susceptibility), and iii) the ability of the host to sustain vector populations [6]–[8].These parameters vary among host species and genotypes [2], [6]–[9], and knowing which factors determine such variation will facilitate identifying the potential of a host as a reservoir for pathogen emergence.

Host lifespan has been identified as a trait related to host reservoir potential. The rationale for linking host lifespan and reservoir potential is that evolution of defenses against pathogens has a fitness cost for the host in terms of other advantageous life history traits, such as fecundity or survival [10]–[13]. Because the probability of becoming infected is higher in long-lived host individuals than in short-lived ones, disease prevalence will be higher in populations of long-lived hosts. Thus, long-lived hosts will be under higher selection pressures for developing costly defenses than short-lived hosts. Model analyses under different scenarios do indeed show that long-lived hosts generally will invest more in defense against pathogens [14]. Major forms of defense are resistance, which has been defined as mechanisms that reduce infection and/or pathogen multiplication in the infected host [15], [16], and tolerance, defined as mechanisms that reduce the negative impact of infection on host fitness, i.e., that reduce pathogen virulence [15], [17]. By the same rationale, longer-lived hosts would be expected to have developed defenses that would reduce their ability to sustain vector populations. However, the relationship between lifespan and defense is a complex one and, because the demographic turnover of short-lived hosts is high and that of long-lived ones is low, it may be influenced by demographic factors, notably by population density and composition [14], [18]. This is in agreement with experimental data showing a link between host density and defense evolution [19]–[21]. Despite the attention received from theoreticians, experimental analyses of the relationship between host lifespan and defense are scarce. For plants, there is evidence that short-lived species of grasses (Poaceae) have a high reservoir potential of a generalist plant virus for long-lived hosts [22], [23]. Also, it has been experimentally shown that defenses reducing susceptibility to infection by that virus was lower in short-lived (annual) that in long-lived (perennial) species of grasses, while competence was not explained by lifespan [8], [24].To our knowledge, the relationship between host lifespan and reservoir potential has not been analyzed at the within-species diversity level.

Here we address the hypothesis that there is a trade-off between defense to a vectored pathogen, and to its vector, and host lifespan, which would explain pathogen reservoir potential. Specifically we ask the following set of questions: i) is host lifespan related to competence, susceptibility and the ability to sustain vector populations? ii) is host lifespan related to reservoir potential?, and iii) does reservoir potential depend on the density and genetic composition of the host population?. For this we used an experimental approach for estimating realistic values of the parameters determining host reservoir potential, as a basis for model analyses of the factors that modulate reservoir potential. We focused our research on a plant-virus system: the wild plant *Arabidopsis thaliana* L. Heynh. (Brassicaceae), its natural pathogen *Cucumber mosaic virus* (*Bromoviridae*), and the virus insect vector *Myzus persicae* Sulzer (*Aphididae*), an interactive assembly of biological components found in nature. *A. thaliana* (from here on, *Arabidopsis*) has been for a long time the model organism of choice for plant molecular genetics, and is increasingly used in analyses of plant ecology, including the evolutionary ecology of plant-pathogen interactions [e.g. 25–29]. *Arabidopsis* is an annual species, presently distributed worldwide after experiencing an expansion from its native range in Eurasia and North Africa [30]. The genetic structure of *Arabidopsis* in the Iberian Peninsula has been studied in detail, demonstrating that it is a centre of genetic diversity for this species [31]–[33]. Demographical analyses carried on in the Iberian Peninsula indicated that *Arabidopsis* plants flower and complete their life cycle in spring, and that populations are built of two or one cohorts of plants that either germinate in the autumn and overwinter as rosettes, or germinate in the spring [29], [34]. Also, populations are genetically heterogeneous and are composed of short-lived genotypes, which do not require vernalization to complete their life cycle, and of long-lived ones, that usually require vernalization and belong to the autumn cohort [32], [35]. *Cucumber mosaic virus* (CMV) is a single-stranded, messenger sense RNA virus, with a three-partite genome encapsidated in isometric particles. CMV has an extremely broad host range, infecting over 1200 species in more than 100 plant families. CMV is efficiently transmitted by more than 75 species of aphids (Hemiptera: *Aphididae*) [36], in a non-persistent, stylet-borne manner, i.e., CMV does not infect the insect vector, instead, particles are retained in the maxillary stylets, allowing the aphid to transmit the virus for a short time (less than 6 h) after acquisition. The green peach aphid (*M. persicae*), a cosmopolitan aphid species, is an important component of the aphid populations feeding on plants in a variety of habitats in Spain (http://www.cabi.org/isc/datasheet/35642), including those in which wild *Arabidopsis* populations are present. *M. persicae* is one of the most efficient vectors for CMV, and is frequently used in transmission experimentation [37]. Analyses of virus infection in six wild *Arabidopsis* populations from different habitats of Central Spain have shown that CMV was the major viral pathogen, with prevalence reaching over 70%, according to the population and the year [29 and unpublished data]. Importantly, while maximum virus prevalence occurred at early developmental stages of the plants, plants remained infected and infectious for the rest of their life. CMV is also transmitted through the seed, with efficiency varying largely according to the plant species and genotype, and it has been reported in *Arabidopsis* to vary between 2 to 8% [38]. It has been shown that *Arabidopsis* genotypes differ in their ability to sustain populations of *M. persicae* [39]. Also, *Arabidopsis* genotypes differ in resistance and tolerance to CMV, and previous work in our group has shown that tolerance to CMV infection was correlated positively with lifespan in *Arabidopsis* [40], [41].

We used an experimental approach comparing two *Arabidopsis* genotypes that differ largely in lifespan, for the following traits: competence as a source for infection, susceptibility to infection, ability to sustain vector populations and rates of seed transmission. These data were then used as a basis to estimate realistic values of the parameters for the analysis of host reservoir potential using a model in which the dynamics of infected plants and of viruliferous vectors were jointly considered. Our results show that the short-lived and the long-lived genotypes differed in susceptibility and in their ability to sustain vector populations, which resulted in a higher reservoir potential for the short-lived host. However, reservoir efficiency of the short-lived genotype was modulated by the density of host plant and vector populations, and by the genetic composition of the host population.

## Materials and Experimental Methods

### Virus and plant material, CMV inoculation and detection

The CMV strain LS-CMV used in this work was derived from biologically active cDNA clones [42] (Accession numbers AF416899, AF416900 and AF127976 for genomic RNAs 1, 2 and 3, respectively). *In vitro* transcripts were multiplied in *Nicotiana clevelandii* plants, virions were purified as in Lot et al. [43] and viral RNA was extracted by virion disruption with phenol and sodium dodecyl sulphate. Two genotypes of *Arabidopsis*, *Landsberg erecta* (Ler) and *Llagostera* (Ll-0), were chosen for the very different span of their life-cycle and tolerance to LS-CMV infection [40], [41]. The genotype *Columbia glabrata 1* (Colgb1) was used as an additional virus source for aphid transmission of CMV to Ler and Ll-0. These three *Arabidopsis* genotypes were initially multiplied simultaneously under the same greenhouse conditions to minimize maternal effects. For experiments, *Arabidopsis* seeds were surface-sterilized, plated on one-half-strength Murashige and Skoog basal salt mix medium [44], 1% (w/v) sucrose, 0.8% (w/v) Bacto agar, and stratified in the dark at 4°C for 96 h. Plates were then transferred to a growth chamber at 22°C under long day conditions (16 h light/8 h dark) and 65–70% relative humidity. Five days-old seedlings were transplanted into 96 well trays with a mix 3∶1, peat∶vermiculite, and after 10 days were transferred into individual 10 cm diameter pots containing the same substrate, in order to minimize spatial and resource limitation. Plants were grown in a growth chamber at 22°C under normal light (220–250 µmol.S^−1^.m^−2^) and long day conditions with 65–70% relative humidity. Generally 2–3 days post-transfer, when plants had 4–5 leaves (stages 1.04–1.05 as in Boyes et *al.* [45]), three rosette leaves per plant were mechanically inoculated with a total of 15 µl of 100 µg/ml suspension of LS-CMV RNA in 0.1M Na_2_HPO_4_. Only 15 µl of 0.1M Na_2_HPO_4_ were applied to mock-inoculated controls. Ten days post-inoculation (dpi), three circles of 4 mm diameter were cut from three randomly chosen systemically infected leaves. In this sample, systemic infection was confirmed by ELISA with PathoScreen CMV Kit (Agdia, Elkhart, IN, USA), and virus accumulation was quantified by quantitative PCR (qRT-PCR). For this, total RNA extracts were obtained using TRIzol reagent according to manufacturer's protocol (Life Technologies, Carlsbad, CA, USA), and then utilized with Brilliant III Ultra-Fast SYBR Green QRT-PCR Master Mix following manufacturer's recommendations (Agilent Technologies, Santa Clara, CA, USA) in a final volume of 10 µl. Assays were performed in triplicate on a LightCycler 480 II real-time PCR system (Roche, Indianapolis, IN, USA). Primers CMV-CP LS Q Fwd (TAAGAAGCTTGTTTCGCGCATTC) and CMV-CP LS Q Rev (CGGAAAGATCGGATGATGAAGG) were designed to amplify 106 nt of the LS-CMV coat protein (CP) gene (GenBank: AF_127976) and primers UBI/PE4Fwd (AATGCTTGGAGTCCTGCTTG) and UBI/PE4 Rev (CTTAGAAGATTCCCTGAGTCGC) amplified 107 nt of the peroxin4 mRNA (GenBank: NM_122477) used as the loading internal control. Quantification was expressed as pg of viral RNA per ng of total RNA. No-template reactions were included in each trial. Thermal parameter for RT-PCR amplification were 50°C for 10 min, 95°C for 3 min and 40 cycles of 95°C for 5 s and 60°C for 10 s. Dissociation curves were generated to ascertain that only one single product was produced and detected in each case.

### Measure of physiological parameters and life-history traits of *Arabidopsis*

Time to bolting and to flowering was estimated as the number of days from the end of stratification until the appearance of the reproductive meristem and the first open flower, respectively. Rosette longevity was estimated as the number of days from the end of stratification until 50% of rosette leaf-senescence. Total plant lifespan was estimated as the time in days from the end of stratification until 50% of siliques had shattered. To quantify leaf mass per unit area (LMA, g×m^−2^), leaves were harvested at flowering and digitally captured for area measurement using the software ImageJ 1.47v (NIH, USA, http://rsbweb.nih.gov/ij/index.html). Then, leaf dry weight was determined after maintaining the leaves at 60°C until constant weight was reached. Rosette relative growth rate (cm/(day×cm)) was estimated by digital capture and further measure of rosette diameter increase every 2 days for a period of 12 days post inoculation. LMA and relative growth rate are parameters that relate to quick-return and slow-return physiological phenotypes, which may influence host competence, susceptibility, and the ability to support vectors [8], [24].

To assay germination potential, the same day as siliques were ripe, seeds were collected and plated onto germination media without surface-sterilization. Seeds were then either directly transferred to a light/dark regime as previously mentioned or they were submitted to stratification prior to the light/dark protocol. The percentage of germinated seeds was determined 4 days post-transfer to the light/dark cycle. To analyze the effect of virus infection on seed germination, a period of 10 months dormancy (storage in the dark at room temperature) was applied to the seeds before carrying out the regular aforementioned surface-sterilization/stratification protocol. Seed transmission of LS-CMV was estimated by qRT-PCR, testing fifteen biological replicates, consisting of a mix of six 5-day-old seedlings. Virus transmission rate for a single seed was then estimated using the expression reported by Gibbs and Gower [46], 
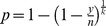
, where *p* is the probability of virus transmission by a single seed, *y* is the number of positive samples, *n* is the total number of samples assayed (*n* = 15), and *k* is the number of seedlings per sample (*k* = 6).

### Evaluation of susceptibility, competence, aphid preference and capacity to sustain aphid vector populations

Experiments were performed using a clonal population of the CMV aphid vector *M. persicae* derived from a single virginiparous apterous female collected in a pepper crop at Alcalá de Henares (Madrid, Spain) in 1989. In order to obtain apterous or alate adult aphids adapted to *Arabidopsis*, two aphid colonies were maintained at ICA-CSIC (Madrid, Spain) (Lat. 40°43′97″N, Long. 3°68′69″W, Alt. 710 m) on an equal mixture of Ler and Ll-0 plants in rearing cages in environmental growth chambers under different conditions: i) For producing apterous aphids, the colony was maintained at low aphid density at 23/18°C (light/dark) temperature, 14 h/10 h (light/dark) photoperiod and 60–80% relative humidity, and ii) Alate aphids were reared at 20/16°C (light/dark) temperature, 12 h/12 h (light/dark) photoperiod and 60–80% relative humidity. Newly emerged alates of 0–48 h of age were used for experiments.

Host plant preference assays were performed with non-viruliferous alate aphids in several dual-choice assays within a 1 m^3^ arena. Aphids were offered as choices either mock inoculated Ler and Ll-0 plants, or LS-CMV-infected Ler and Ll-0 plants, or mock-inoculated and LS-CMV infected plants of either genotype. Twenty plants, ten of each choice class, were randomly placed in each arena during the trial. Two hundred winged aphids were placed in a flight platform similar to the one described by Fereres et al. [47] and released 0.5 m above the test plants. Settled alate individuals, as well as nymphs produced on each plant, were counted 24 h after their release.

To estimate the capacity of mock-inoculated and LS-CMV-infected Ler and Ll-0 plants to sustain vector populations, the intrinsic rate of natural increase of aphids was determined. Apterous adults of the *M. persicae* colony of equal age and weight were used. For synchronization, three apterous adult females were placed on each *Arabidopsis* test plant and confined using a plastic cup. The following day, adults were removed and three newly born nymphs were kept on each plant, which reached adulthood 7–8 days later. At that point, only one adult was kept per test plant, and every 24 h the newly born nymphs were counted and removed to avoid crowding effects that could influence the reproductive potential. The intrinsic rate of natural increase of the aphid population (r_m_) was calculated according to the equation proposed by Wyatt and White [48], 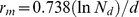, where *N_d_* is the number of progeny produced by an adult during *d* days, *d* being the pre-reproductive period (number of days from birth to first reproduction). Between 25 and 34 plants per treatment, for a total of 116 plants, were used in this study.

Transmission assays were performed as described by Fereres et al. [49]. Briefly, groups of 30–40 apterous individuals of *M. persicae* were collected and after a pre-acquisition starvation period of one hour were released on LS-CMV infected source plants. All infected plants used as sources for transmission exhibited a saturated optical density (OD) in an ELISA test for CMV. Following a 10 min acquisition access period, groups of 5 aphids were transferred to each of the 3 week-old *Arabidopsis* target plants. A 24 h inoculation access period was allowed before spraying plants with imidacloprid (Confidor, Bayer). Plants were then transferred to an aphid-free growth chamber at 22°C under long day cycle. Three weeks post-inoculation, plants were tested by ELISA for LS-CMV infection. Samples were considered positive when their OD was 3 times higher than the negative control's OD after 24 h of incubation. A total of 37 source plants were sequentially tested, with transmission assayed to 252 Ler and 252 Ll-0 target plants. Transmission rate by a single aphid vector was estimated based on the Gibbs and Gower equation [46] (see above), from the number of positive samples (*y*), the total number of samples assayed (*n*), and the number of aphids used per transmission trial (*k* = 5).

All the above experiments involving *M. persicae* were performed twice, and results are presented as the mean values of both experiments.

### Statistical analyses

Statistical analyses were performed using the statistical software package Statgraphics Centurion version 15.1.02 (StatPoint technologies, Inc., Warrenton, VA). The data sets were analyzed using analysis of variance (ANOVA) and transformation by 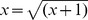 was applied when necessary for data normalization.

## Experimental Results

### Characterization of phenotypic traits of *Arabidopsis thaliana* genotypes

The two genotypes of *Arabidopsis* used in this work, Ler and Ll-0, presented large differences in their life history. Lifespan (from germination until senescence, see Material & Methods) of Ler plants was of 57.25±0.70 days (d), while it was significantly longer, 87.88±1.06 d, for Ll-0 (Table 1) (F_1,16_ = 581.15, *P*≤10^−5^). Thus, lifespan defined a short-lived (Ler) and a long-lived (Ll-0) genotype. Differences in lifespan between Ler and Ll-0 were also associated with differences in each of any other temporal life-history trait measured (F_1,16_≥234.37, *P*≤10^−5^), such as time to bolting, flowering time or time to first silique shattered (Table 1). In addition to differing in temporal life-history traits, Ler and Ll-0 plants also differed broadly in morphology. As reported [40], [41] the allometric relationship between vegetative (rosette) and reproductive (inflorescence) parts was much larger for Ll-0, which had large rosette built of more than 150 leaves, as compared with the small rosettes of Ler plants, with less than 10 leaves (Figure S1). Also growth rate estimated by rosette diameter increase per day was higher in Ll-0 than in Ler, but the leaf mass per unit area did not differ between both genotypes (Table 1).

**Table 1:** Phenotypic traits of two *Arabidopsis* genotypes1).

	*Landsberg erecta* (Ler)		Llagostera (Ll-0)	
	mock	CMV-LS infected	mock	CMV-LS infected
Lifespan (days)	57.25±0.70 c	58.50±0.50 c	87.88±1.06 b	99.25±2.19 a
Bolting (days)	17.88±0.23 b	17.88±0.23 b	46.25±1.84 a	46.00±2.91 a
Flowering (days)	22.38±0.18 c	23.13±0.30 c	52.38±1.74 b	57.38±2.66 a
Silique shattered (days)	37.75±0.31 c	39.75±0.53 c	68.25±1.90 b	75.38±2.71 a
Rosette senescence (days)	43.75±2.32 c	45.88±0.69 c	74.75±0.49 b	79.75±1.95 a
Rosette relative growth rate [cm/(day×cm)]	0.12±0.02ab	0.11±0.02 b	0.16±0.01 a	0.11±0.01 b
Leaf Mass/Area (g/m^2^)	19.83±0.24 a	20.35±0.79 a	19.12±1.18 a	18.23±1.53 a
Seed germination (%)2)	76.41±7.34 a	78.65±6.25 a	78.88±3.63 a	76.34±5.16 a

1) Data are mean ± standard error of 8 plants except for rosette growth (5 plants) and leaf mass per unit area (3 plants with 17 (Ler) or 30 (Ll-0) leaves per plant on average). For each trait, values followed by different letters are significantly different in an ANOVA analysis.

2) After a 10 month dormancy period.

Ler and Ll-0 plants also differed in seed germination potential. While seed germination rate was similar for Ler and Ll-0 after a ten month dormancy period (Table 1), seeds of Ler had a germination rate of 95% as soon as siliques reached maturity. This was not the case for Ll-0, which seeds showed a germination rate of 0% at silique maturation. These data indicate a lack of stratification or dormancy requirements for Ler seeds, and the need of a dormancy period for Ll-0 seed germination.

### Susceptibility of *Arabidopsis thaliana* genotypes to LS-CMV infection under different transmission modes

To test if lifespan was related to LS-CMV multiplication in Ler and Ll-0 genotypes, virus accumulation in systemically infected leaves ten dpi was analyzed. Virus accumulation did not differ significantly in Ler and in Ll-0 plants (5.35±1.74 pg/ng and 2.57±0.28 pg/ng, respectively,F_1,16_ = 2.48, *P* = 0.1376).

Next, we tested host susceptibility to horizontal transmission, i.e., the probability of the host becoming infected by a viruliferous *M. persicae* vector. Transmission experiments were performed using Ler and Ll-0 plants as targets for transmission, and Ler, Ll-0 and Colgb1 plants as sources for virus acquisition by aphids. Data (Table 2) show that Ler and Ll-0 plants were similarly susceptible to LS-CMV aphid transmission when the aphids acquired the virus in plants of the same genotype (9.67±2.89 and 8.72±1.91% transmission for Ler and Ll-0, respectively, F_1,74_ = 0.59, *P* = 0.4431). Nonetheless, susceptibility to transmission in Ll-0 plants was dependent on the inoculum source, being significantly lower (F_2,37_ = 4.35, *P* = 0.0208) when aphids acquired the virus in Ler plants (3.83±0.85%) than when the virus was acquired in Ll-0 or in Colgb1 plants (8.72±1.91% and 10.48±2.13%, respectively). On the contrary, susceptibility of Ler plants was not affected by the genotype of the plants in which the virus was acquired (F_2,37_ = 0.28, *P* = 0.7548).

**Table 2:** Transmission rates of CMV-LS by a single *Myzus persicae* from three infected *Arabidopsis* genotype sources to Ler or Ll-01).

Target plants	Source for aphid acquisition of LS-CMV		
	*Columbia glabrata 1*	*Landsberg erecta* (Ler)	Llagostera (Ll-0)
Ler	9.03±1.63 a	9.67±2.89 a	7.31±2.03 a
Ll-0	10.48±2.13 a	3.83±0.85 b	8.72±1.91 a

1) Transmission rates were calculated as in [46]. Data are mean ± standard error for at least 10 source plants. Values followed by different letters are different in an ANOVA test.

Last, susceptibility to vertical transmission through the seed was similar for Ler and Ll-0 plants, the rate of single seed transmission being of 1.98±0.70 and 2.47±0.82%, respectively (values from eight assayed mother plants, F_1,16_ = 0.21, *P* = 0.6537).

### Effect of CMV infection on host plant traits

The effects of LS-CMV infection differed in the two *Arabidopsis* genotypes analyzed (Table 1). Infection resulted in an increase of the lifespan of Ll-0 plants to 99.25±2.19 d, in comparison with mock-inoculated plants (F_1,16_ = 22.07, *P* = 0.0003), while the lifespan of infected Ler plants did not differ from that of mock-inoculated plants (58.5±0.5 days, F_1,16_ = 2.11, *P* = 0.1685). The longer life-span of infected Ll-0 plants is the result of reduced growth rates. Thus, infection resulted in a reduction of the relative rate of rosette growth for Ll-0 plants (F_1,10_ = 10.53, *P* = 0.0118) but not for Ler plants (F_1,10_ = 0.11, *P* = 0.7476), and in a delay in the development of the inflorescence, as shown by the time to flowering and to silique maturation (Table 1). Neither the leaf mass per unit area (F_1,6_≤0.41, *P*≥0.5587) or the seed germination rate after a 10 month dormancy (F_1,16_≤0.16, *P*≥0.6930) were affected by infection in either genotype.

### Vector preference and host capacity to sustain vector populations

Two parameters related to host competence, i.e., to the host capacity as a source of virus for vector transmission, were also estimated for Ler and Ll-0 plants: their capacity to attract vectors and to sustain vector populations.

Results from dual free-choice experiments showed that a similar number of winged morphs of *M. persicae* had settled on mock-inoculated Ler and Ll-0 plants, and a similar number of nymphs were produced in both genotypes after 24 h, which is an indication of the time an aphid spends on the plant on which it has landed (Table 3, Cage 3, F_1,40_ = 0.92, *P* = 0.3424 and F_1,40_ = 1.47, *P* = 0.2333 respectively). When mock-inoculated and infected plants were compared, it was found that more aphids settled on mock-inoculated than on LS-CMV-infected plants of both genotypes (Table 3, Cage 1 and Cage 2). While this preferential settlement was only marginally significant for each plant genotype, Ler or Ll-0, when analyzed separately (F_1,40_ = 1.72, *P* = 0.1981 and F_1,40_ = 3.37, *P* = 0.0743 for Ler and Ll-0 respectively), it was significant when data from both genotypes were pooled together (F_1,80_ = 4.86, *P* = 0.0304), indicating that virus infection reduced aphid preference after 24 h. Similarly, more nymphs were produced in mock-inoculated plants than in infected ones (F_1,40_ = 1.92, *P* = 0.1738 and F_1,40_ = 9.20, *P* = 0.0043 for Ler and Ll-0 plants, respectively, the difference being significant when data from both genotypes were pooled, F_1,80_ = 4.40, *P* = 0.0391). Interestingly, more winged adult aphids were recovered from infected Ler than from infected Ll-0 plants (Table 3, Cage 4; F_1,40_ = 4.53, *P* = 0.0398), and those winged adults had produced more nymphs after 24 h (F_1,40_ = 6.98, *P* = 0.0119).

**Table 3:** *Myzus persicae* adults and nymphs recovered 24 h post release on two different genotypes of *Arabidopsis* 1).

	Cage 1			Cage 2			Cage 3			Cage 4		
	Ler			Ll-0			Mock			CMV-LS infected		
	Mock	CMV-LS infected	*P*	Mock	CMV-LS infected	*P*	Ler	Ll-0	*P*	Ler	Ll-0	*P*
No. insects settled/plant	5.33±1.22	3.42±0.80	0.1981	5.48±0.93	3.27±0.76	0.0743	5.95±1.68	4.00±1.13	0.3424	5.90±0.96	3.50±0.59	0.0398
No nymphs/plant	6.36±1.64	3.64±1.07	0.1738	6.80±1.12	2.76±0.73	0.0043	6.24±1.70	3.76±1.13	0.2333	6.73±1.16	3.27±0.61	0.0119
% recovery	79.40±5.83 ab			86.90±7.42 ab			94.97±0.03 a			73.51±4.76 b		

1) Data are mean ± standard error of 20 plants per treatment. Comparisons for No. insects settled per plant ad No. nymphs per plants were done within each cage, and *P* value for each character is indicated (ANOVA test). Comparisons for percentage of aphid recovery are done among cages, and values followed by different letters are different in an ANOVA test.

We also estimated the intrinsic rate of natural increase (*r_m_*), and other reproduction-related parameters, of the *M. persicae* population on mock-inoculated and LS-CMV-infected plants of Ler and Ll-0 genotypes. Results in Table 4 show that the aphid's pre-reproductive period was affected neither by plant genotype nor by infection status (F_3,116_ = 0.99, *P* = 0.3996). On the other hand, a trend was observed towards higher numbers of total nymphs produced, daily fecundity and *r_m_* from adults feeding on infected plants as compared with those from mock-inoculated plants. Although this trend was not statistically significant for all parameters (see Table 4), it was consistent for both genotypes (F_1,53_≥2.52, *P*≤0.1182 and F_1,63_≥2.37, *P*≤0.1290 for Ll-0 and Ler, respectively) and statistically significant when data from both genotypes were pooled together (F_1,116_≥5.41, *P*≤0.0217). Interestingly, when plants were considered according to genotype, and not to infection status, it was found that Ler plants were able to better support aphid population growth than Ll-0 plants, as *r_m_* values differed significantly (0.3697 vs. 0.3506, F_1,116_ = 3.89, *P* = 0.0509).

**Table 4:** Parameters of the life cycle of *Myzus persicae* grown on two different genotypes of *Arabidopsis* 1).

	Ler		Ll-0	
	Mock	CMV-LS infected	Mock	CMV-LS infected
Pre-eproductive period (days)	7.55±0.09 a	7.50±0.09 a	7.72±0.09 a	7.57±0.10 a
Total nymphs/adult	41.51±1.36 ab	45.08±1.15 a	40.38±1.54 b	42.85±1.44 ab
Daily fecundity/adult	5.52±0.19 b	6.03±0.15 a	5.24±0.20 b	5.66±0.17 ab
Intrinsic rate of increase (*r_m_*)	0.3642±0.0056 ab	0.3752±0.0046 a	0.3531±0.0055 b	0.3660±0.00485 ab

1) Data are mean ± standard error of, at least, 25 aphids. For each character, values followed by different letters are different in an ANOVA test.

## Model Methods

To analyze the differences between short- and long-lived genotypes of *Arabidopsis* in reservoir potential, and the effects of host plant density and population composition in this trait, we used a model in which the dynamics of the plant and vector populations were jointly considered, based on that proposed by Madden et al. [50]. In this model, the rate of plant infection depends on the interaction of susceptible non-infected plants with infectious vectors, hence on the density of infected plants and infectious vectors. Our model differs from that of Madden *et al.* [50] in that: i) We considered the plant population divided into two classes, susceptible non-infected (*S*), and infected (*I*). We did not consider a separated class of infected-non-infectious plants (i.e. we do not consider a latent period) since the rate of colonization of host tissues and organs by the virus, which determines transmissibility, is not genotype-dependent (our unpublished results). Neither did we consider a class of recovered plants, as CMV causes systemic chronic infections so that plants, once infected, remain so until the end of their life cycle. Also, infected plants were considered to be a source of infection for aphids until the end of their life cycle, because aphids fed on stalks and cauline leaves until senescence (our unpublished observations). ii) The aphid vector population was divided into two classes, non-viruliferous (*X*), and viruliferous aphids (*Z*), i.e., aphids that had acquired the virus by feeding on infected, *I*, plants and were able to transmit it by feeding on non-infected, *S*, plants. Since CMV is transmitted non-persistently, there is no latent period for transmission. iii) A major difference with Madden *et al.* [50] model is that virulence, ν, expressed as the effect of infection on plant mortality) was considered in our model. For a single host, the dynamics of the model is described by the following equations:
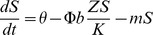
(1)

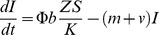
(2)

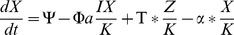
(3)

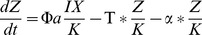
(4)These equations represent the variation with time (days) of the density of non-infected susceptible plants, *S* (Eq. 1), of infected plants, *I*, (Eq. 2), both expressed as plants×m^−2^, and of virus-free aphids, *X*, (Eq. 3) and viruliferous aphids, *Z*, (Eq. 4), expressed as aphids×plant^−1^. *K* indicates the maximum density of the plant population. Parameter *m* indicates the *per capita* plant mortality rate, and parameter *v* indicates the variation of plant mortality rate due to infection (i.e., virulence). The transmission rate is decomposed into two parameters, *Φ*, which indicates the number of susceptible plants visited per day by an aphid, and *b*, which represents the probability of virus transmission per vector visit. Similarly, parameter *a* indicates the probability that a vector acquires the virus at each visit. Because transmission is non-persistent, viruliferous vectors lose the virus with a rate τ, returning to the *X* class of virus-free vectors. Last, α indicates the per capita mortality of aphids, which is not affected by their viruliferous state.

This model was extended to two hosts and its dynamics for Host 1 are described by the set of equations:
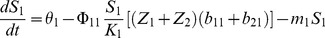
(5)

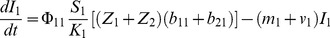
(6)

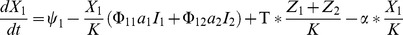
(7)

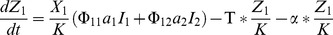
(8)Subscripts 1 and 2 denote the host. Equations 5–8 describe the variation with time (days) of the density of susceptible plants, *S_i_* (Eq. 5), of infected plants, *I_i_*, (Eq. 6), expressed as plants×m^−2^, and of virus-free aphids, *X_i_*, (Eq. 7) and viruliferous aphids, *Z_i_*, (Eq. 8), expressed as aphids×plant^−1^, *i* = (1,2), and the model differs from the single host model (Eq. 1–4) in allowing for aphids to visit Host 2 from Host 1 (parameter *Φ_12_*), from which acquisition of virus and transmission to Host 1 from Host 2 occurred (parameter *b*_21_). A similar set of equations describes the dynamics for Host 2 (not shown); the only differences would be the substitution subscript numbers 2 for 1 and *vice versa*.

Definitions and values of model parameters are shown in Table 5, in which Host 1 and Host 2 represent a short-lived and a long-lived genotype, respectively. Accordingly, model parameters were estimated from the experimental data obtained for Ler and Ll-0, respectively, as in the previous sections. Because in *Arabidopsis* CMV infection is not lethal, virulence (*ν_i_*) was estimated as the variation of the plant's lifespan by infection, *D_I_*, as compared with the lifespan of uninfected susceptible plants *D_S_*, following Day [51]. Mortality rates relate to lifespan by *m* = 1/*D_S_*, for non-infected plants, and (*m*+*ν_i_*) = 1/*D_Ii_* for infected plants. Data in Table 1 show that CMV infection does not affect the lifespan of Ler, while it delays the completion of Ll-0 life cycle, resulting in a negative virulence. Note that the lifespan of susceptible non-infected plants in Table 5 is shorter than the lifespan of mock-inoculated plants in Table 1, as we considered that plants would not attract aphids until they had reached the four-leaf stage. Similarly, the mortality rate of aphids, α, was estimated as the inverse of their lifespan, which was of an average of 40 d in the conditions in which experiments were performed. Transmission probability by single aphids during each visit of an S plant from an I plant (parameter *b*) was according to the data in Table 2. Following Madden et al. [50] we considered that the probability of acquisition of a non-persistently transmitted virus (parameter *a*) is the same as the probability of transmission, and that the aphid remains viruliferous for a maximum of 6 h (parameter τ). The frequency of aphid visits to S plants from I plants was not estimated experimentally, and was given arbitrary values varying between 0.01 and 1. These values may be realistic, because epidemiological studies of CMV in different regions of Spain for different years indicate transmission rates of 0.008–0.122 days^−1^ [52]. However, because mock inoculated and infected plants of genotypes Ler and Ll-0 showed different vector preference, the probability of aphid visits from infected Ll-0 to healthy Ler plants was considered to be 0.63 times the probability of visits from infected Ler to healthy Ler plants, infected Ll-0 to healthy Ll-0 or infected Ler to healthy Ll-0 plants, according to the data in Table 3.

**Table 5:** Parameter values for model simulations.

Parameter	Definition	Value for Host 1 (Short-lived)	Value for Host 2 (Long-lived)
D_S_	Lifespan of S plants	42 days (d)	70 d
m = 1/D_S_	Mortality rate of S plants	0.0238 d^−1^	0.0143 d^−1^
D_I_	Lifespan of I plants	42 d	81 d
v	Virulence: (m+v) = 1/D_I_	0	−0.0019 d^−1^
b_ii_	Probability of transmission to S_i_ from S_i_	0.0967	0.0731
b_ji_	Probability of transmission to S_i_ from S_j_	0.0872	0.0383
Φ_ii_	Rate at which a vector goes from an S to an I plant of the same host	Φ_11_ = Φ	Φ_22_ = Φ
Φ_ji_	Rate at which a vector goes from an S to an I plant of the other host	Φ_21_ = Φ	Φ_12_ = 0.63Φ
1/τ	Infectious period of vector	0.25 d^−1^	0.25 d^−1^
a	Prob. of aphids acquiring the virus ^b^	0.0967	0.0731
D_x_	Lifespan of aphids	40 d	40 d
α = 1/D_x_	Mortality rate of aphids	0.025 d^−1^	0.025 d^−1^
*K*	maximum density of the plant population	model tested with 5≤*K*≤500 for both hosts	
*Q*	maximum density of the insect vector population	model tested with 0.1≤*Q*≤5 for both hosts	
*Φ*	rate at which a vector goes from a host to another	model tested with 0.01≤*Φ*≤1 for both hosts.	

For simulations, we considered a monomolecular growth of the population of susceptible plants, *θ* = *r_p_* (*K*−*T*), where *T = S+I*, is the total plant population density, *K* is the maximum density of the population, and *r_p_* is its growth rate. To make the plant population constant during a growing season, we set *r_p_* = 1 to get a constant value of *T* = *K*. Simulations were performed for 5<*K*<500, according to data on the variation of plant density in wild *Arabidopsis* populations in Central Spain over sites and years [29, and unpublished results]. Similarly, we made the aphid population constant by considering its growth, ψ as monomolecular according to *ψ = r_a_ (Q−P)/K*, where *P* = *X*+*Z* is the total aphid population, *Q* is its maximum density, and r_a_ = 1 for a constant aphid population density per plant. *Q* was made to vary between 0.1 and 5 aphids per plant, which are realistic values of density of aphid populations in Central Spain [53]. For all simulations the initial condition was that 2% of the plants were CMV infected (i.e., values of *I_1_* and *I_2_* of 0.02*K_i_*), according to the experimentally determined rates of vertical transmission of LS-CMV in *Arabidopsis* (this work). All simulations were done in R.

## Model Simulation Results

**Figure 1:**
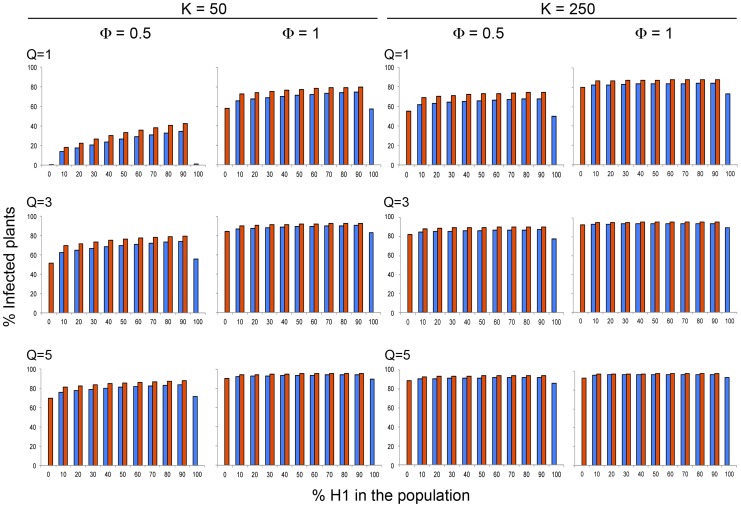
Model simulation values of the variation of CMV incidence in each of the two *Arabidopsis* genotypes, a short-lived (Host 1 in blue) and a long-lived (Host 2 in red) genotype, within a heterogeneous *Arabidopsis* population according to the relative frequency of each genotype in the population. Data are presented for two different host plant population densities (*K*, plants×m^−2^), three different aphid densities (*Q*, aphids×plant^−1^) and two different rates of visit per aphids (*Φ*, visits×day^−1^) at equilibrium.

For all model simulations the frequency of short-lived Host 1 and of the long-lived Host 2 in the plant population was made to vary between 0 and 100%. The threshold values of the density of the aphid population, *Q*, and of the probability of aphid visits to healthy plants from infected ones, *Φ*, necessary for the occurrence of a CMV epidemic, depended on the total plant population density (*K*). For *K* = 5 epidemics occurred at *Φ*≥1 and *Q*≥3, while for *K* = 50 and *K* = 100 epidemics occurred for *Φ*≥0.5 and *Q*≥1, and for *K* = 250 or higher, epidemics occurred for *Φ*≥0.1 and *Q*≥3, or for *Φ*≥0.5 and *Q*≥1 (Fig. 1 and Table S1). Hence, plant and aphid population densities are primary factors determining CMV infection. Note that although *Φ* was made to vary independently of *Q*, it is known that aphid mobility increases as aphid population density increases [50], so that in a real situation *Φ* would not be independent of *Q*.

The incidence of CMV (i.e., the fraction of infected plants, *I*) at equilibrium was compared first for single-genotype populations of either the short-lived Host 1 or the long-lived Host 2. It was found that incidence was higher for the long-lived host for a broad range of values of plant and aphid population density (*K*, and *Q* values) and of probability of aphid visits to non-infected plants from infected ones (*Φ* values). Incidence was higher for the short-lived host only for plant and aphid densities, and probability of aphid visits to plants, that would result in low transmission rates, e.g., for any *Φ* and *Q* value if *K* = 5, and for low *Φ* and *Q* values for higher *K*, e.g., *Φ* = 0.5 if *K* = 50, *Φ* = 0.5 if *Q* = 1 when *K* = 100, or *Φ* = 0.1 for *Q* = 5 or *Q*≥3 when *K* = 250 or *K* = 500, respectively (Fig. 1 and Table S1). Hence, the highest competence and capacity to sustain aphid populations of the short-lived genotype is countered by the longer infectious period of the long-lived one when host and/or aphids are abundant and transmission is more effective.

**Figure 2:**
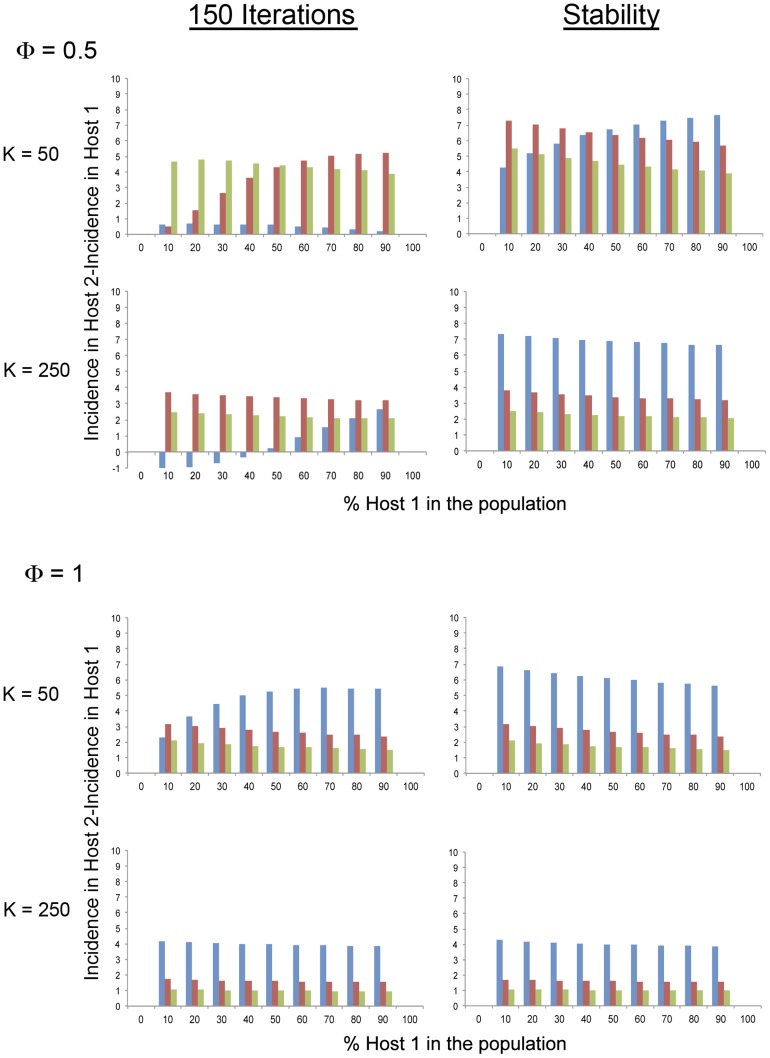
Model simulation values of the variation of the difference between CMV incidence in a long-lived (Host 2) and a short-lived (Host 1) *Arabidopsis* genotype, within a heterogeneous population according to the relative frequency of each genotype in the population. Data are presented for two different host plant population densities (*K*, plants×m^−2^), three different aphid densities (*Q*, aphids×plant^−1^, *Q* = 1 in blue, *Q* = 3 in red, *Q* = 5 in green) and two different rates of visit per aphids (*Φ*, visits×day^−1^), both after 150 iterations of the model and at equilibrium.

Then, CMV incidence at equilibrium was compared for populations that were built of mixtures of short- and long-lived hosts in different proportions. For heterogeneous host populations, model simulation analyses showed that for any values of plant and aphid population density, and of the probability of aphid visits to non-infected plants from infected ones, *K*,, *Q*, and *Φ*, the total incidence in the plant population, and the incidence for each plant genotype, was always higher than for single-host genotype populations (Fig. 1, and Table S1). This result indicates that when the population is heterogeneous, there is a balance between the different epidemiological parameters, such as transmission rate, vector preference or infectious period, that result in higher incidence. However, for all explored conditions, the total incidence increased with increasing frequency of the short-lived host in the plant population, and incidence was always higher for the long-lived than for the short-lived host (Fig. 1 and Table S1). These two results indicate that the short-lived host was always a better source of infection for the long-lived one than *vice versa*. Thus, for all analyzed conditions, the short-lived host was a more efficient reservoir than the long-lived one. The difference between CMV incidence in the long-lived and short-lived hosts, however, depended on the density of the aphid population, *Q*, and their mobility, *Φ*, so that for any plant density value the difference increased with increasing *Q* if *Φ*≥1, but had a minimum at *Q* = 3 for low *Φ* values (e.g., 0.5). Thus, the efficiency as a reservoir of the short-lived host depended non-linearly on the rates of transmission (Fig. 1 and Table S1). The difference between CMV incidence in the long- and short-lived hosts also depended on the density and genetic composition of the host population: for *K* = 5, the difference had relative maxima for different frequencies of the short-lived host according to the value of *Q*; for *K* = 50, *Φ* = 0.5 and *Q* = 1, the difference in CMV incidences in both hosts increased monotonically with the frequency of the short-lived host in the population. For any other values of *K*, *Φ* and *Q*, the difference between incidences in the long- and short-lived hosts decreased monotonically with the frequency of the short-lived host in the population (see Fig. 2 for examples). Thus, the efficiency of the short-lived host as a reservoir depended in a complex non-linear way on the genetic composition of the host population, modulated by the density of the host and vector populations and by the rate of aphid mobility, hence modulated by the factors determining transmission efficiency.

We considered next the values of CMV incidence in each host, and of their difference, after 150 iterations, which informs about the evolution of the epidemics before equilibrium. Also, this is a temporal frame more in line with the *Arabidopsis* life cycle of about 5 months. After 150 iterations the data (Fig. 2 and Table S2) show a more dramatic variation of CMV incidence according to values of plant and aphid density and aphid mobility, and according to the frequency of the short-lived host in the population, than at equilibrium. Notably, the difference between incidences in the long- and short-lived hosts showed relative maxima for a wider range of values of plant and aphid density and aphid mobility. The difference could even show negative values, either with relative minima or monotonically increasing with increasing proportion of the short-lived host in the population, depending on the density of the host and vector populations. It is interesting to underscore that when plant population density was high (high values of *K*), and both aphid population density and aphid mobility were low (low values of *Q* and *Φ*), and the proportion of the short-lived host in the population was low, the incidence in the short-lived host was higher than in the long-lived one. This result indicates that under this set of conditions the epidemic progressed faster in the short-lived host at earlier times, and faster in the long-lived host at later times. Thus, this result underlies the role of the short-lived host as a reservoir.

## Discussion

Vectored viruses comprise a large fraction of emerging pathogens of humans, animals and plants, and important research efforts are devoted to understand the network of ecological and evolutionary factors that determine virus emergence [2]–[5], [54]. Generally, emerging pathogens can be permanently maintained in reservoir hosts [2], [3], and identification of the determinants of host reservoir potential is central to understand emergence and infection dynamics. Within this context, the relationship between host lifespan and reservoir potential is an understudied field. It has been proposed that long-lived hosts will invest more in costly defenses against pathogens because they are more exposed to infection than short-lived hosts. Consequently, short-lived hosts will be more susceptible to infection, more competent as sources of infection and/or will sustain larger vector populations, hence being effective reservoirs for the infection of long-lived hosts [8], [14], [55]. Evidence for a link between host lifespan and reservoir potential derives mostly from the comparison of species within the host range of a pathogen [6], [7], [22], [23], [56]–[58], and the underlying mechanisms have rarely been analyzed [e.g. 8,24,58]. To our knowledge, the relationship between host lifespan, defense and reservoir potential, has not been analyzed at the intra-species level, in spite of abundant evidence of genetic diversity resulting in polymorphisms for lifespan and defense within species.

It has been shown that tolerance to CMV infection in *A. thaliana* correlates positively with lifespan, the longer-lived genotypes showing a higher tolerance to CMV infection [40], [41]. Higher tolerance was, at least in part, due to the ability of long-lived genotypes to modify their developmental schedule upon infection, so that more resources were allocated to reproduction than to growth [40], [41]. In this study we focus on two *Arabidopsis* genotypes that represent extremes of lifespan and tolerance, i.e., Ler and Ll-0 [40], [41], as representatives of the short- and long-lived genotypes that co-exist in wild *Arabidopsis* populations in different regions of the Iberian Peninsula [29], [32], [35]. Ler and Ll-0 plants differed sharply in the temporal parameters of their life-cycle, in their morphology and in other relevant life-history traits (Table 1 and [40], [41]). Among these, it is noteworthy that seeds of the short-lived genotype, Ler, did not require a period of dormancy before germination, which will allow them to have more than one generation per growing season under field conditions [29], [34]. This will not be the case for the long-lived Ll-0-like genotypes that may require a period of dormancy for germination and vernalization for flowering. CMV infection did not affect germination rate of any genotype tested, and CMV vertical transmission rate did not differ for the short- and long-lived genotypes. Thus, it is tempting to speculate that CMV will follow different strategies to increase its fitness in short- and long-lived genotypes. In the long-lived genotype, which has only one generation per year, infection increases the lifespan (i.e., the infectious period), which is not modified in the short-lived genotype, which may have more than one generation per year. Interestingly, in this system we cannot equate short and long lifespan with the physiological phenotypes of Quick- or Slow-Return, since rosette relative growth rate was higher in the long-lived Ll-0 genotype than in the short-lived Ler, but leaf mass per unit area did not differ between both genotypes. This unexpected result is at odds with evidence derived from the comparison of different plant species [8], [24], [59], [60], and suggests that the trade-off between lifespan and development and reproductive rates [61] may not apply at the level of within-species diversity, or at the temporal scale at which the lifespan of Ler and Ll-0 differ. The different performance of aphids in each genotype, regardless of similar LMA, could be explained by differences in nutrient composition in the phloem sap [62], which would not translate into detectable differences in LMA.

Using these two *Arabidopsis* genotypes we analyzed if lifespan, in addition to correlating positively with tolerance to CMV [41], also correlated with susceptibility, competence and the ability to sustain vector populations; three parameters that determine reservoir potential [8]. Ler and Ll-0 plants were similarly competent sources of infection, as well as similarly susceptible to infection when transmission assays were performed between plants of the same genotype. These results agree with the non-significant difference in virus accumulation between Ler and Ll-0 plants, transmission rates of CMV in different hosts having been shown to increase with virus titer until saturation at high virus titers [63], [64]. On the other hand, the susceptibility to infection of Ll-0, but not of Ler, depended on the genotype of the plant from which the aphid vector acquired the virus. Hence, the susceptibility of the long-lived genotype will be, on the average, lower in a heterogeneous plant population than that of the short-lived genotype. The capacity to sustain aphid vector populations was higher in the short-lived Ler genotype than in the long-lived Ll-0 one, when both mock-inoculated and CMV-infected plants were considered. In addition, more aphids landed and settled, and more nymphs were generated, in CMV-infected, but not in mock-inoculated, Ler than in Ll-0 plants, indicating that the capacity to sustain vector populations was differentially modified for each genotype by virus infection, and was higher in the short-lived genotype. It is noteworthy that the results of our aphid host preference experiments agree with previous reports involving different host plant species infected with CMV, in which it was shown that after an initial attraction to CMV-infected plants, aphids migrated and settled into mock-inoculated ones at some time between 30 and 60 min after landing [65], [66]. All these results taken together indicate that two of the three epidemiological parameters related to reservoir potential, susceptibility to infection and capacity to sustain vector populations, were higher in the short-lived than in the long-lived genotype, which would indicate a higher reservoir potential of the short-lived genotype. Thus, our results agree with predictions on the relationship between host lifespan and defense evolution [14], [55] and with evidence derived from the comparison of different hosts of multi-host parasites infecting both plants and animals [6]–[8], [56]–[68]. It is important to point-out that most previous evidence for a relationship between lifespan, defense and reservoir potential, was derived from the comparison of different species with large differences in lifespan, of the order of years, e.g., rodent species with life expectancies of 2 vs. 4–8 years, or annual vs. perennial plant species [8], [56]. Our results extend that evidence to a much narrower time-scale in lifespan differences, in the order of weeks, as expected for the variability in lifespan at the within species level for a short-lived plant. Although our experimental results derive from the study of only one short- and one long-lived genotype, they indicate that a link between lifespan and defense at the within-species level will result in polymorphisms for reservoir potential within a heterogeneous single-species host population. There is growing evidence for covariance between host traits determining reservoir potential and local extinction risk [55]. If longer lived, less susceptible and competent host genotypes are also at higher local extinction risk is a question to be explored, as it could determine both disease dynamics and the evolution of apparently unrelated traits in host populations.

In terms of reservoir efficiency, the lower defenses, i.e., higher susceptibility and capacity to sustain vector populations, of the short-lived *Arabidopsis* genotype could be balanced by the longer infectious period of the long-lived genotype, and the balance might be modulated by the demography and genetic composition of the host population. To explore how the efficiency as a reservoir is affected by host demography and by the genetic composition of the host population, we developed a simple epidemiological model which is essentially a simplification of that proposed by Madden *et al.* [50] with the addition of a virulence parameter to take into account the effect of infection on host plant mortality. The model was run for a set of realistic parameters derived from the above-discussed experiments with Ler and Ll-0, and for a wide range of host plant and aphid population densities according to field observations [29], [53]. The negative virulence in the long-lived Host 2 deserves some consideration. The negative virulence value result from the increase in lifespan of long-lived *Arabidopsis* genotypes upon infection, which associated with resource reallocation resulting in tolerance [41], and is linked to a decrease in growth rate (Table 1). Under field conditions in which plants will compete, the slower-growing infected long-lived plants could be at a competitive disadvantage, hence suffering from a positive virulence as was shown in experiments in which plant density was manipulated [21]. Model simulation analyses were done considering different virulence values, the negative virulence value shown in Table 5, virulence 0, or a positive virulence of the same amount, with no substantial difference in the results. Thus, even in the case that negative virulence were an artifact of green-house experimentation, it will not affect the outcome of model simulation analyses.

When the model was run for single-genotype populations of either short-lived or long-lived hosts, it was found that the short-lived host population sustained higher incidence of CMV only at low plant and/or aphid populations densities, i.e., under conditions of low transmission rates. Thus, the higher reservoir potential of short-lived genotypes due to lower defense was partly countered by the longer infectious period of long-lived genotypes when transmission was highly efficient (i.e. higher *K*, *Q* and *Φ*). In agreement with this result, we found that the equilibrium incidence of CMV was always higher for mixed-genotype host populations than for single-genotype populations of either short- or long-lived hosts, underscoring the role in determining the reservoir efficiency of both host defense and infectious period. The total CMV incidence in mixed-genotype populations increased with increasing frequency of the short-lived genotype, and incidence was always higher in the long-lived genotype subpopulation, indicating that for the realistic parameters and within the wide range of conditions considered, the short-lived genotype is always a better reservoir than the long-lived one. How good a reservoir was the short-lived genotype, however, depended in a complex way on the density of the host and aphid populations, as determinants of the probability of transmission. These non-linear effects were more pronounced when we analyzed the predictions of the models after 150 iterations, which may approximate the *Arabidopsis* growth period, rather than the equilibrium values. After 150 iterations, the difference between CMV incidence in long- and short-lived hosts showed maxima, or even became negative, for a given *K* value according to the *Q* and *Φ* values (Fig. 2 and Table S2). Data from 150 iterations also showed that the relative rate of epidemic growth in each host varied with time. The complex relationship found between efficiency as a reservoir of the short-lived genotype, the density of host and aphid populations, and the genetic composition of the host population, is in agreement both with model predictions on the variation of defense with host population traits [14], [18] and with our experimental results with the same host-pathogen system [21].

In summary, in this work we show that the hypothesis stating that there is a correlation between host lifespan and investment in defenses against pathogens, developed and tested for the different hosts of multi-host pathogens, also holds for two genotypes of a single host species, which may differ in lifespan at much smaller temporal scales. Analyses of more short- and long-lived genotypes would be required for generalization, but our results indicate that the less efficient defenses of short-lived genotypes result in their higher reservoir potential. However, the reservoir potential of short-lived genotypes may be balanced in heterogeneous host populations by the longer infectious period of long-lived genotypes. Model simulations under realistic parameter ranges showed that this balance is modulated according to complex, non-linear relations, by the demography of both the host and vector populations, and by the genetic composition of the host population, an important conclusion that often is not considered in analyses of the evolutionary ecology of pathogen emergence. Thus, within-species genetic diversity for lifespan and defenses against pathogens will result in polymorphisms for pathogen reservoir potential, which will condition within-population infection dynamics. These results should be taken into account in the future in joint analyses of the population genetics of traits determining host defense and lifespan, to get a better understanding of the evolution of defense against pathogens in host populations.

## Supporting Information

Figure S1 **Morphological differences among the short-lived and long-lived Arabidopsis genotypes Ler and Ll-0 along their development.** The white bar on the left of each image indicates 5 cm.
(TIF)

Table S1 **Model simulation values of the density of infected plants of a short-lived and a long-lived host genotype (Host 1 and Host 2, respectively) in a heterogeneous host population at equilibrium, for different values of K, Φ and Q.**
(DOCX)

Table S2 **Model simulation values of the density of infected plants of a short-lived and a long-lived host genotype (Host 1 and Host 2, respectively) in a heterogeneous host population after 150 iterations, for different values of K, Φ and Q.**
(DOCX)
