# Supplementary figures and images for: The Relationship between Host Lifespan and Pathogen Reservoir Potential: An Analysis in the System Arabidopsis thaliana-Cucumber mosaic virus
Source: PLoS Pathog. 2014 Nov 6;10(11):e1004492. doi: 10.1371/journal.ppat.1004492 (PMC4223077; doi:10.1371/journal.ppat.1004492)

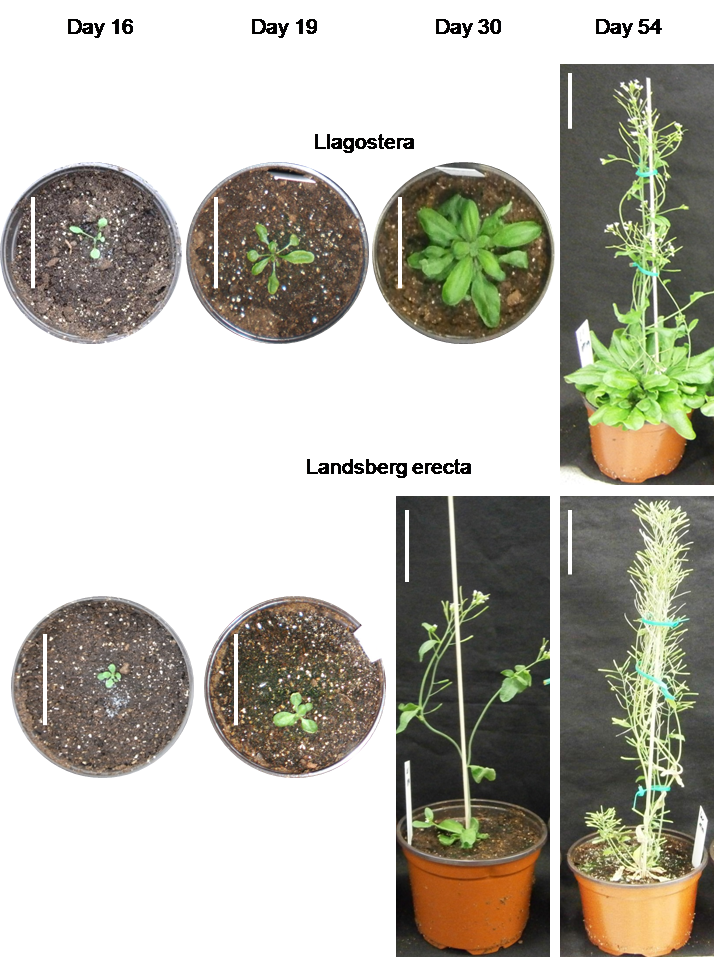

Supplement: Figure S1 — Morphological differences among the short-lived and long-lived Arabidopsis genotypes Ler and Ll-0 along their development. The white bar on the left of each image indicates 5 cm. (TIF) [file ppat.1004492.s001.tif]
