# Supplementary material for: The Relationship between Host Lifespan and Pathogen Reservoir Potential: An Analysis in the System Arabidopsis thaliana-Cucumber mosaic virus
Source: PLoS Pathog. 2014 Nov 6;10(11):e1004492. doi: 10.1371/journal.ppat.1004492 (PMC4223077; doi:10.1371/journal.ppat.1004492)
Supplement: Table S1 — Model simulation values of the density of infected plants of a short-lived and a long-lived host genotype (Host 1 and Host 2, respectively) in a heterogeneous host population at equilibrium, for different values of K, Φ and Q. (DOCX) [file ppat.1004492.s002.docx]

Table S1. Model simulation values of the density of infected plants of a short-lived and a long-lived host genotype (Host 1 and Host 2, respectively) in a heterogeneous host population at equilibrium, for different values of K, Φ and Q.

|  |  |  | **H1 = 0 H2 = 100** | | **H1 =10**  **H2 =90** | | **H1 =20**  **H2 = 80** | | **H1 = 30**  **H2 = 70** | | **H1 = 40**  **H2 = 60** | | **H1 = 50**  **H2 = 50** | | **H1 = 60**  **H2 = 40** | | **H1 = 70**  **H2 = 30** | | **H1 = 80**  **H2 = 20** | | **H1 = 90**  **H2 = 10** | | **H1 = 100**  **H2 = 0** | |
| --- | --- | --- | --- | --- | --- | --- | --- | --- | --- | --- | --- | --- | --- | --- | --- | --- | --- | --- | --- | --- | --- | --- | --- | --- |
| **K** | **φ** | **Q** | **I1** | **I2** | **I1** | **I2** | **I1** | **I2** | **I1** | **I2** | **I1** | **I2** | **I1** | **I2** | **I1** | **I2** | **I1** | **I2** | **I1** | **I2** | **I1** | **I2** | **I1** | **I2** |
| **5** | 0.1 | 1 | - | - | - | - | - | - | - | - | - | - | - | - | - | - | - | - | - | - | - | - | - | - |
|  |  | 3 | - | - | - | - | - | - | - | - | - | - | - | - | - | - | - | - | - | - | - | - | - | - |
|  |  | 5 | - | - | - | - | - | - | - | - | - | - | - | - | - | - | - | - | - | - | - | - | - | - |
|  |  |  |  |  |  |  |  |  |  |  |  |  |  |  |  |  |  |  |  |  |  |  |  |  |
|  | 0.5 | 1 | - | - | - | - | - | - | - | - | - | - | - | - | - | - | - | - | - | - | - | - | - | - |
|  |  | 3 | - | - | - | - | - | - | - | - | - | - | - | - | - | - | - | - | - | - | - | - | - | - |
|  |  | 5 | - | - | - | - | - | - | - | - | - | - | - | - | - | - | - | - | - | - | ~~-~~ | ~~-~~ | - | - |
|  |  |  |  |  |  |  |  |  |  |  |  |  |  |  |  |  |  |  |  |  |  |  |  |  |
|  | 1 | 1 | - | - | ~~-~~ | ~~-~~ | - | - | ~~-~~ | ~~-~~ | - | - | ~~-~~ | ~~-~~ | - | - | ~~-~~ | ~~-~~ | - | - | ~~-~~ | ~~-~~ | - |  |
|  |  | 3 | - | 0.02 | 0.16 | 1.77 | 0.32 | 1.56 | 0.53 | 1.51 | 0.78 | 1.41 | 1.05 | 1.25 | 1.35 | 1.06 | 1.66 | 0.83 | 1.99 | 0.58 | 2.88 | 0.35 | 0.76 | - |
|  |  | 5 | - | 1.74 | 0.3 | 3 | 0.56 | 2.55 | 0.88 | 2.33 | 1.23 | 2.07 | 1.6 | 1.77 | 1.97 | 1.45 | 2.36 | 1.11 | 2.76 | 0.75 | 3.66 | 0.42 | 2.36 | - |
|  |  |  |  |  |  |  |  |  |  |  |  |  |  |  |  |  |  |  |  |  |  |  |  |  |
| **50** | 0.1 | 1 | - | - | - | - | **~~-~~** | **~~-~~** | **~~-~~** | **~~-~~** | **~~-~~** | **~~-~~** | **~~-~~** | **~~-~~** | **~~-~~** | **~~-~~** | **~~-~~** | **~~-~~** | **~~-~~** | **~~-~~** | **~~-~~** | **~~-~~** | **~~-~~** | **~~-~~** |
|  |  | 3 | - | - | - | - | **~~-~~** | **~~-~~** | **~~-~~** | **~~-~~** | **~~-~~** | **~~-~~** | **~~-~~** | **~~-~~** | **~~-~~** | **~~-~~** | **~~-~~** | **~~-~~** | **~~-~~** | **~~-~~** | **~~-~~** | **~~-~~** | **~~-~~** | **~~-~~** |
|  |  | 5 | - | - | - | - | **~~-~~** | **~~-~~** | **~~-~~** | **~~-~~** | **~~-~~** | **~~-~~** | **~~-~~** | **~~-~~** | **~~-~~** | **~~-~~** | **~~-~~** | **~~-~~** | **~~-~~** | **~~-~~** | **~~-~~** | **~~-~~** | **~~-~~** | **~~-~~** |
|  |  |  |  |  |  |  |  |  |  |  |  |  |  |  |  |  |  |  |  |  |  |  |  |  |
|  | 0.5 | 1 | - | 0.01 | 0.68 | 8.04 | 1.73 | 8.99 | 3.1 | 9.26 | 4.72 | 8.98 | 6.57 | 8.26 | 8.61 | 7.15 | 10.81 | 5.73 | 13.13 | 4.03 | 15.55 | 2.11 | 0.44 | - |
|  |  | 3 | - | 25.74 | 3.11 | 31.28 | 6.46 | 28.65 | 9.99 | 25.69 | 13.67 | 22.47 | 17.45 | 19.04 | 21.32 | 15.45 | 25.26 | 11.73 | 29.24 | 7.9 | 33.27 | 3.98 | 27.79 | - |
|  |  | 5 | - | 34.81 | 3.79 | 36.58 | 7.75 | 33.06 | 11.83 | 29.32 | 16.01 | 25.42 | 20.26 | 21.38 | 24.56 | 17.24 | 28.9 | 13.01 | 33.28 | 8.73 | 37.68 | 4.38 | 35.72 | - |
|  |  |  |  |  |  |  |  |  |  |  |  |  |  |  |  |  |  |  |  |  |  |  |  |  |
|  | 1 | 1 | - | 29 | 3.3 | 32.78 | 6.78 | 29.76 | 10.39 | 26.49 | 14.11 | 23.04 | 17.9 | 19.43 | 21.76 | 15.7 | 25.66 | 11.87 | 29.59 | 7.97 | 33.55 | 4.01 | 28.85 | - |
|  |  | 3 | - | 42.23 | 4.36 | 40.66 | 8.79 | 36.38 | 13.28 | 32.01 | 17.81 | 27.55 | 22.38 | 23.05 | 26.96 | 18.49 | 31.56 | 13.9 | 36.18 | 9.29 | 40.8 | 4.65 | 41.67 | - |
|  |  | 5 | - | 45.23 | 4.6 | 42.36 | 9.26 | 37.8 | 13.94 | 33.18 | 18.66 | 28.52 | 23.39 | 23.81 | 28.14 | 19.09 | 32.89 | 14.34 | 37.66 | 9.57 | 42.43 | 4.79 | 44.81 | - |
|  |  |  |  |  |  |  |  |  |  |  |  |  |  |  |  |  |  |  |  |  |  |  |  |  |

|  |  |  | **H1 = 0 H2 = 100** | | **H1 =10**  **H2 =90** | | **H1 =20**  **H2 = 80** | | **H1 = 30**  **H2 = 70** | | **H1 = 40**  **H2 = 60** | | **H1 = 50**  **H2 = 50** | | **H1 = 60**  **H2 = 40** | | **H1 = 70**  **H2 = 30** | | **H1 = 80**  **H2 = 20** | | **H1 = 90**  **H2 = 10** | | **H1 = 100**  **H2 = 0** | |
| --- | --- | --- | --- | --- | --- | --- | --- | --- | --- | --- | --- | --- | --- | --- | --- | --- | --- | --- | --- | --- | --- | --- | --- | --- |
| **K** | **φ** | **Q** | **I1** | **I2** | **I1** | **I2** | **I1** | **I2** | **I1** | **I2** | **I1** | **I2** | **I1** | **I2** | **I1** | **I2** | **I1** | **I2** | **I1** | **I2** | **I1** | **I2** | **I1** | **I2** |
| **100** | 0.1 | 1 | - | - | - | - | - | - | - | - | - | - | - | - | - | - | - | - | - | - | - | - | - | - |
|  |  | 3 | - | - | - | - | - | - | - | - | - | - | - | - | - | - | - | - | - | - | - | - | - | - |
|  |  | 5 | - | - | - | - | - | - | - | - | - | - | - | - | - | - | - | - | - | - | - | - | - | - |
|  |  |  |  |  |  |  |  |  |  |  |  |  |  |  |  |  |  |  |  |  |  |  |  |  |
|  | 0.5 | 1 | - | 26.82 | 4.23 | 45.28 | 8.96 | 42.29 | 14.09 | 38.54 | 19.55 | 34.16 | 25.27 | 29.27 | 31.18 | 23.97 | 37.25 | 18.34 | 43.44 | 12.43 | 49.72 | 6.31 | 31.28 | - |
|  |  | 3 | - | 70.54 | 7.59 | 73.19 | 15.45 | 65.95 | 23.51 | 58.35 | 31.72 | 50.47 | 40.04 | 42.38 | 48.45 | 34.12 | 56.93 | 25.73 | 65.45 | 17.23 | 74 | 8.65 | 69.46 | - |
|  |  | 5 | - | 81.56 | 8.48 | 79.65 | 17.14 | 71.37 | 25.92 | 62.85 | 34.81 | 54.16 | 43.77 | 45.33 | 52.78 | 36.4 | 61.83 | 27.38 | 70.92 | 18.3 | 80.01 | 9.17 | 80.36 | - |
|  |  |  |  |  |  |  |  |  |  |  |  |  |  |  |  |  |  |  |  |  |  |  |  |  |
|  | 1 | 1 | - | 71.59 | 7.6 | 73.23 | 15.39 | 65.75 | 23.33 | 58 | 31.38 | 50.05 | 39.5 | 41.94 | 47.69 | 33.71 | 55.91 | 25.38 | 64.17 | 16.97 | 72.43 | 8.51 | 67.31 | - |
|  |  | 3 | - | 89.49 | 9.1 | 84.03 | 18.29 | 74.94 | 27.54 | 65.74 | 36.82 | 56.47 | 46.14 | 47.15 | 55.48 | 37.77 | 64.83 | 28.36 | 74.19 | 18.93 | 83.56 | 9.47 | 87.12 | - |
|  |  | 5 | - | 93.55 | 9.45 | 86.37 | 18.95 | 76.92 | 28.49 | 67.41 | 38.05 | 57.86 | 47.63 | 48.27 | 57.23 | 38.65 | 66.83 | 29.01 | 76.44 | 19.35 | 86.06 | 9.68 | 91.98 | - |
|  |  |  |  |  |  |  |  |  |  |  |  |  |  |  |  |  |  |  |  |  |  |  |  |  |
| **250** | 0.1 | 1 | - | - | - | - | ~~-~~ | ~~-~~ | ~~-~~ | ~~-~~ | ~~-~~ | ~~-~~ | ~~-~~ | ~~-~~ | ~~-~~ | ~~-~~ | ~~-~~ | ~~-~~ | ~~-~~ | ~~-~~ | ~~-~~ | ~~-~~ | ~~-~~ |  |
|  |  | 3 | - | - | 0.01 | 0.14 | 0.04 | 0.23 | 0.11 | 0.38 | 0.29 | 0.63 | 0.71 | 1 | 1.61 | 1.5 | 3.39 | 1.99 | 6.48 | 2.2 | 11.17 | 1.67 | ~~-~~ |  |
|  |  | 5 | - | 0.23 | 3.12 | 36.78 | 8.36 | 43.3 | 15.28 | 45.66 | 23.51 | 44.71 | 32.83 | 41.23 | 43.05 | 35.75 | 54.04 | 28.64 | 65.65 | 20.16 | 77.75 | 10.55 | 3 |  |
|  |  |  |  |  |  |  |  |  |  |  |  |  |  |  |  |  |  |  |  |  |  |  |  |  |
|  | 0.5 | 1 | - | 138.14 | 15.46 | 155.59 | 31.55 | 140.56 | 48.12 | 124.64 | 65.04 | 108.01 | 82.23 | 90.83 | 99.62 | 73.22 | 117.14 | 55.26 | 134.76 | 37.03 | 152.41 | 18.59 | 125.04 | - |
|  |  | 3 | - | 204.97 | 21.14 | 198.74 | 42.57 | 177.59 | 64.23 | 156.06 | 86.03 | 134.24 | 107.96 | 112.19 | 129.96 | 89.96 | 152.01 | 67.6 | 174.1 | 45.14 | 196.2 | 22.6 | 194.46 | - |
|  |  | 5 | - | 221.81 | 22.58 | 208.81 | 45.35 | 186.2 | 68.26 | 163.34 | 91.27 | 140.3 | 114.36 | 117.12 | 137.49 | 93.83 | 160.66 | 70.45 | 183.84 | 47.01 | 207.03 | 23.52 | 214.29 | - |
|  |  |  |  |  |  |  |  |  |  |  |  |  |  |  |  |  |  |  |  |  |  |  |  |  |
|  | 1 | 1 | - | 199.38 | 20.54 | 194.49 | 41.31 | 173.58 | 62.23 | 152.38 | 83.26 | 130.96 | 104.38 | 109.37 | 125.54 | 87.65 | 146.73 | 65.83 | 167.92 | 43.93 | 189.11 | 21.98 | 182.7 | - |
|  |  | 3 | - | 231.28 | 23.36 | 214.1 | 46.8 | 190.58 | 70.31 | 166.94 | 93.86 | 143.22 | 117.43 | 119.43 | 141.03 | 95.6 | 164.63 | 71.73 | 188.24 | 47.84 | 211.84 | 23.93 | 223.49 | - |
|  |  | 5 | - | 231.51 | 23.99 | 218.37 | 48.04 | 194.27 | 72.12 | 170.1 | 96.24 | 145.87 | 120.37 | 121.61 | 144.51 | 97.32 | 168.65 | 73.01 | 192.8 | 48.69 | 216.95 | 24.35 | 233.45 | - |
|  |  |  |  |  |  |  |  |  |  |  |  |  |  |  |  |  |  |  |  |  |  |  |  |  |

|  |  |  | **H1 = 0 H2 = 100** | | **H1 =10**  **H2 =90** | | **H1 =20**  **H2 = 80** | | **H1 = 30**  **H2 = 70** | | **H1 = 40**  **H2 = 60** | | **H1 = 50**  **H2 = 50** | | **H1 = 60**  **H2 = 40** | | **H1 = 70**  **H2 = 30** | | **H1 = 80**  **H2 = 20** | | **H1 = 90**  **H2 = 10** | | **H1 = 100**  **H2 = 0** | |
| --- | --- | --- | --- | --- | --- | --- | --- | --- | --- | --- | --- | --- | --- | --- | --- | --- | --- | --- | --- | --- | --- | --- | --- | --- |
| **K** | **φ** | **Q** | **I1** | **I2** | **I1** | **I2** | **I1** | **I2** | **I1** | **I2** | **I1** | **I2** | **I1** | **I2** | **I1** | **I2** | **I1** | **I2** | **I1** | **I2** | **I1** | **I2** | **I1** | **I2** |
|  |  |  |  |  |  |  |  |  |  |  |  |  |  |  |  |  |  |  |  |  |  |  |  |  |
| **500** | 0.1 | 1 | - | - | - | - | - | - | - | - | - | - | - | - | - | - | - | - | - | - | - | - | - | - |
|  |  | 3 | - | 4.5 | 1.71 | 17.93 | 3.28 | 15.4 | 4.8 | 13.2 | 6.34 | 11.24 | 8.01 | 9.45 | 9.9 | 7.75 | 12.12 | 6.05 | 14.82 | 4.26 | 18.14 | 2.29 | 28.58 | - |
|  |  | 5 | - | 134.13 | 4.08 | 38.94 | 7.86 | 33.5 | 11.58 | 28.88 | 15.52 | 24.86 | 19.94 | 21.23 | 25.17 | 17.76 | 31.59 | 14.2 | 39.63 | 10.28 | 49.84 | 5.68 | 156.47 | - |
|  |  |  |  |  |  |  |  |  |  |  |  |  |  |  |  |  |  |  |  |  |  |  |  |  |
|  | 0.5 | 1 | - | 323.7 | 34.43 | 339.07 | 69.55 | 303.8 | 105.19 | 267.55 | 141.19 | 230.55 | 177.47 | 192.95 | 213.91 | 154.91 | 250.47 | 116.51 | 287.06 | 77.85 | 323.65 | 38.99 | 281.35 | - |
|  |  | 3 | - | 429.08 | 43.76 | 407.92 | 87.84 | 363.59 | 132.14 | 318.83 | 176.61 | 273.77 | 221.18 | 228.47 | 265.83 | 182.99 | 310.51 | 137.36 | 355.2 | 91.64 | 399.88 | 45.84 | 402.83 | - |
|  |  | 5 | - | 455.58 | 46.1 | 424.04 | 92.4 | 377.54 | 138.85 | 330.79 | 185.4 | 283.83 | 232.02 | 236.73 | 278.68 | 189.51 | 325.37 | 142.21 | 372.06 | 94.85 | 418.74 | 47.44 | 437.53 | - |
|  |  |  |  |  |  |  |  |  |  |  |  |  |  |  |  |  |  |  |  |  |  |  |  |  |
|  | 1 | 1 | - | 412.38 | 42.15 | 396.56 | 84.54 | 353.26 | 127.1 | 309.64 | 169.77 | 265.77 | 212.52 | 221.72 | 255.3 | 177.52 | 298.09 | 133.22 | 340.86 | 88.85 | 383.58 | 44.43 | 375.06 | - |
|  |  | 3 | - | 467.59 | 47.11 | 430.9 | 94.32 | 383.31 | 141.6 | 335.59 | 188.91 | 287.79 | 236.26 | 239.91 | 283.62 | 191.98 | 330.98 | 144.01 | 378.33 | 96.02 | 425.66 | 48.01 | 450.78 | - |
|  |  | 5 | - | 480.12 | 48.23 | 438.37 | 96.52 | 389.84 | 144.85 | 341.23 | 193.21 | 292.57 | 241.59 | 243.86 | 289.97 | 195.12 | 338.36 | 146.36 | 386.73 | 97.58 | 435.1 | 48.79 | 469.34 | - |
