# Supplementary material for: The Relationship between Host Lifespan and Pathogen Reservoir Potential: An Analysis in the System Arabidopsis thaliana-Cucumber mosaic virus
Source: PLoS Pathog. 2014 Nov 6;10(11):e1004492. doi: 10.1371/journal.ppat.1004492 (PMC4223077; doi:10.1371/journal.ppat.1004492)
Supplement: Table S2 — Model simulation values of the density of infected plants of a short-lived and a long-lived host genotype (Host 1 and Host 2, respectively) in a heterogeneous host population after 150 iterations, for different values of K, Φ and Q. (DOCX) [file ppat.1004492.s003.docx]

Table S2. Model simulation values of the density of infected plants of a short-lived and a long-lived host genotype (Host 1 and Host 2, respectively) in a heterogeneous host population after 150 iterations, for different values of K, Φ and Q.

|  |  |  | **H1 = 0 H2 = 100** | | **H1 =10**  **H2 =90** | | **H1 =20**  **H2 = 80** | | **H1 = 30**  **H2 = 70** | | **H1 = 40**  **H2 = 60** | | **H1 = 50**  **H2 = 50** | | **H1 = 60**  **H2 = 40** | | **H1 = 70**  **H2 = 30** | | **H1 = 80**  **H2 = 20** | | **H1 = 90**  **H2 = 10** | | **H1 = 100**  **H2 = 0** | |
| --- | --- | --- | --- | --- | --- | --- | --- | --- | --- | --- | --- | --- | --- | --- | --- | --- | --- | --- | --- | --- | --- | --- | --- | --- |
| **K** | **φ** | **Q** | **I1** | **I2** | **I1** | **I2** | **I1** | **I2** | **I1** | **I2** | **I1** | **I2** | **I1** | **I2** | **I1** | **I2** | **I1** | **I2** | **I1** | **I2** | **I1** | **I2** | **I1** | **I2** |
| **5** | 0.1 | 1 | - | - | - | - | - | - | - | - | - | - | - | - | - | - | - | - | - | - | - | - | - | - |
|  |  | 3 | - | - | - | - | - | - | - | - | - | - | - | - | - | - | - | - | - | - | - | - | - | - |
|  |  | 5 | - | - | - | - | - | - | - | - | - | - | - | - | - | - | - | - | - | - | - | - | - | - |
|  |  |  |  |  |  |  |  |  |  |  |  |  |  |  |  |  |  |  |  |  |  |  |  |  |
|  | 0.5 | 1 | - | - | - | - | - | - | - | - | - | - | - | - | - | - | - | - | - | - | - | - | - | - |
|  |  | 3 | - | - | - | - | - | - | - | - | - | - | - | - | - | - | - | - | - | - | - | - | - | - |
|  |  | 5 | - | - | - | - | - | - | - | - | - | - | - | - | - | - | - | - | - | - | ~~-~~ | ~~-~~ | - | - |
|  |  |  |  |  |  |  |  |  |  |  |  |  |  |  |  |  |  |  |  |  |  |  |  |  |
|  | 1 | 1 | - | 0.02 | 0 | 0.04 | 0 | 0.03 | 0.01 | 0.03 | 0.01 | 0.03 | 0.01 | 0.02 | 0.02 | 0.02 | 0.02 | 0.02 | 0.03 | 0.01 | 0.03 | 0.01 | 0.01 | - |
|  |  | 3 | - | 0.09 | 0.03 | 0.28 | 0.08 | 0.33 | 0.16 | 0.39 | 0.29 | 0.45 | 0.49 | 0.49 | 0.76 | 0.51 | 1.1 | 0.47 | 1.5 | 0.38 | 1.92 | 0.22 | 0.21 | - |
|  |  | 5 | - | 0.29 | 0.15 | 1.36 | 0.41 | 1.65 | 0.75 | 1.81 | 1.14 | 1.79 | 1.54 | 1.64 | 1.94 | 1.39 | 2.34 | 1.09 | 2.75 | 0.74 | 3.16 | 0.38 | 1.74 | - |
|  |  |  |  |  |  |  |  |  |  |  |  |  |  |  |  |  |  |  |  |  |  |  |  |  |
| **50** | 0.1 | 1 |  | - | - | - | ~~-~~ | ~~-~~ | ~~-~~ | ~~-~~ | ~~-~~ | ~~-~~ | ~~-~~ | ~~-~~ | ~~-~~ | ~~-~~ | ~~-~~ | ~~-~~ | ~~-~~ | ~~-~~ | ~~-~~ | ~~-~~ | ~~-~~ |  |
|  |  | 3 |  | - | - | - | ~~-~~ | ~~-~~ | ~~-~~ | ~~-~~ | ~~-~~ | ~~-~~ | ~~-~~ | ~~-~~ | ~~-~~ | ~~-~~ | ~~-~~ | ~~-~~ | ~~-~~ | ~~-~~ | ~~-~~ | ~~-~~ | ~~-~~ |  |
|  |  | 5 |  | - | - | - | ~~-~~ | ~~-~~ | ~~-~~ | ~~-~~ | ~~-~~ | ~~-~~ | ~~-~~ | ~~-~~ | ~~-~~ | ~~-~~ | ~~-~~ | ~~-~~ | ~~-~~ | ~~-~~ | ~~-~~ | ~~-~~ | ~~-~~ |  |
|  |  |  |  |  |  |  |  |  |  |  |  |  |  |  |  |  |  |  |  |  |  |  |  |  |
|  | 0.5 | 1 |  | 0.74 | 0.14 | 1.55 | 0.34 | 1.63 | 0.63 | 1.69 | 1.02 | 1.72 | 1.56 | 1.71 | 2.28 | 1.62 | 3.21 | 1.44 | 4.39 | 1.13 | 5.85 | 0.66 | 0.9 |  |
|  |  | 3 |  | 6.74 | 2.37 | 21.55 | 5.53 | 22.73 | 9.17 | 22.32 | 13.04 | 20.64 | 17.01 | 18.09 | 21.03 | 14.97 | 25.07 | 11.5 | 29.13 | 7.8 | 33.2 | 3.95 | 23.63 |  |
|  |  | 5 |  | 22.56 | 3.74 | 35.75 | 7.71 | 32.75 | 11.81 | 29.21 | 16 | 25.37 | 20.25 | 21.36 | 24.56 | 17.23 | 28.9 | 13.01 | 33.28 | 8.73 | 37.68 | 4.38 | 35.59 |  |
|  |  |  |  |  |  |  |  |  |  |  |  |  |  |  |  |  |  |  |  |  |  |  |  |  |
|  | 1 | 1 |  | 11.45 | 3.02 | 28.22 | 6.49 | 27.42 | 10.18 | 25.32 | 13.96 | 22.45 | 17.81 | 19.13 | 21.7 | 15.55 | 25.63 | 11.81 | 29.58 | 7.94 | 33.55 | 4 | 27.74 |  |
|  |  | 3 |  | 42.08 | 4.36 | 40.66 | 8.79 | 36.38 | 13.28 | 32.01 | 17.81 | 27.55 | 22.38 | 23.05 | 26.96 | 18.49 | 31.56 | 13.9 | 36.18 | 9.29 | 40.8 | 4.65 | 41.68 |  |
|  |  | 5 |  | 45.24 | 4.6 | 42.36 | 9.26 | 37.8 | 13.94 | 33.18 | 18.66 | 28.52 | 23.39 | 23.81 | 28.14 | 19.09 | 32.89 | 14.34 | 37.66 | 9.57 | 42.43 | 4.79 | 44.82 |  |

|  | |  | |  | |  | |  | |  | |  | |  | |  | |  | |  | |  | |  | |  | |  | |  | |  | |  | |  | |  | |  | |  |  | |  |  | |
| --- | --- | --- | --- | --- | --- | --- | --- | --- | --- | --- | --- | --- | --- | --- | --- | --- | --- | --- | --- | --- | --- | --- | --- | --- | --- | --- | --- | --- | --- | --- | --- | --- | --- | --- | --- | --- | --- | --- | --- | --- | --- | --- | --- | --- | --- | --- | --- |
|  |  | |  | | **H1 = 0 H2 = 100** | | | | **H1 =10**  **H2 =90** | | | | **H1 =20**  **H2 = 80** | | | | **H1 = 30**  **H2 = 70** | | | | **H1 = 40**  **H2 = 60** | | | | **H1 = 50**  **H2 = 50** | | | | **H1 = 60**  **H2 = 40** | | | | **H1 = 70**  **H2 = 30** | | | | **H1 = 80**  **H2 = 20** | | | | **H1 = 90**  **H2 = 10** | | | **H1 = 100**  **H2 = 0** | | | |
| **K** | **φ** | | **Q** | | **I1** | | **I2** | | **I1** | | **I2** | | **I1** | | **I2** | | **I1** | | **I2** | | **I1** | | **I2** | | **I1** | | **I2** | | **I1** | | **I2** | | **I1** | | **I2** | | **I1** | | **I2** | | **I1** | | **I2** | **I1** | | | **I2** |
| **100** | 0.1 | | 1 | | - | | - | | - | | - | | - | | - | | - | | - | | - | | - | | - | | - | | - | | - | | - | | - | | - | | - | | - | | - | - | | | - |
|  |  | | 3 | | - | | - | | - | | - | | - | | - | | - | | - | | - | | - | | - | | - | | - | | - | | - | | - | | - | | - | | - | | - | - | | | - |
|  |  | | 5 | | - | | - | | - | | - | | - | | - | | - | | - | | - | | - | | - | | - | | - | | - | | - | | - | | - | | - | | - | | - | - | | | - |
|  |  | |  | |  | |  | |  | |  | |  | |  | |  | |  | |  | |  | |  | |  | |  | |  | |  | |  | |  | |  | |  | |  |  | | |  |
|  | 0.5 | | 1 | |  | | 4.01 | | 1.16 | | 10.66 | | 2.93 | | 11.78 | | 5.48 | | 12.66 | | 8.89 | | 13.11 | | 13.23 | | 12.96 | | 18.46 | | 12.06 | | 24.48 | | 10.32 | | 31.13 | | 7.7 | | 38.25 | | 4.24 | 9.89 | | |  |
|  |  | | 3 | |  | | 41.27 | | 7.32 | | 69.35 | | 15.18 | | 64.15 | | 23.29 | | 57.49 | | 31.56 | | 50.06 | | 39.93 | | 42.19 | | 48.38 | | 34.03 | | 56.89 | | 25.69 | | 65.45 | | 17.22 | | 74.03 | | 8.65 | 68.41 | | |  |
|  |  | | 5 | |  | | 76.27 | | 8.47 | | 79.52 | | 17.13 | | 71.32 | | 25.92 | | 62.83 | | 34.81 | | 54.15 | | 43.77 | | 45.33 | | 52.79 | | 36.4 | | 61.85 | | 27.39 | | 70.94 | | 18.31 | | 80.05 | | 9.18 | 80.49 | | |  |
|  |  | |  | |  | |  | |  | |  | |  | |  | |  | |  | |  | |  | |  | |  | |  | |  | |  | |  | |  | |  | |  | |  |  | | |  |
|  | 1 | | 1 | |  | | 54.27 | | 7.53 | | 71.98 | | 15.34 | | 65.21 | | 23.29 | | 57.76 | | 31.36 | | 49.94 | | 39.49 | | 41.89 | | 47.69 | | 33.69 | | 55.93 | | 25.37 | | 64.2 | | 16.97 | | 72.5 | | 8.51 | 67.25 | | |  |
|  |  | | 3 | |  | | 89.53 | | 9.11 | | 84.05 | | 18.3 | | 74.95 | | 27.54 | | 65.75 | | 36.82 | | 56.48 | | 46.14 | | 47.15 | | 55.48 | | 37.77 | | 64.84 | | 28.37 | | 74.21 | | 18.93 | | 83.58 | | 9.47 | 87.19 | | |  |
|  |  | | 5 | |  | | 93.59 | | 9.45 | | 86.38 | | 18.95 | | 76.93 | | 28.49 | | 67.42 | | 38.05 | | 57.86 | | 47.64 | | 48.27 | | 57.23 | | 38.65 | | 66.84 | | 29.01 | | 76.45 | | 19.35 | | 86.07 | | 9.68 | 92.02 | | |  |
|  |  | |  | |  | |  | |  | |  | |  | |  | |  | |  | |  | |  | |  | |  | |  | |  | |  | |  | |  | |  | |  | |  |  | | |  |
| **250** | 0.1 | | 1 | | - | | - | | - | | - | | ~~-~~ | | ~~-~~ | | ~~-~~ | | ~~-~~ | | ~~-~~ | | ~~-~~ | | ~~-~~ | | ~~-~~ | | ~~-~~ | | ~~-~~ | | ~~-~~ | | ~~-~~ | | ~~-~~ | | ~~-~~ | | ~~-~~ | | ~~-~~ | ~~-~~ | | |  |
|  |  | | 3 | | - | | - | | 0.32 | | 4.24 | | 0.69 | | 3.95 | | 1.1 | | 3.63 | | 1.57 | | 3.27 | | 2.09 | | 2.86 | | 2.68 | | 2.4 | | 3.33 | | 1.89 | | 4.05 | | 1.32 | | 4.85 | | 0.69 | ~~-~~ | | |  |
|  |  | | 5 | | - | | 7.34 | | 0.73 | | 8.32 | | 1.62 | | 8.01 | | 2.69 | | 7.6 | | 3.95 | | 7.05 | | 5.44 | | 6.35 | | 7.17 | | 5.5 | | 9.17 | | 4.45 | | 11.47 | | 3.2 | | 14.08 | | 1.73 | 4.43 | | |  |
|  |  | |  | |  | |  | |  | |  | |  | |  | |  | |  | |  | |  | |  | |  | |  | |  | |  | |  | |  | |  | |  | |  |  | | |  |
|  | 0.5 | | 1 | | - | | 28.9 | | 8.29 | | 72.39 | | 19.56 | | 76.34 | | 33.52 | | 76.93 | | 49.59 | | 73.93 | | 67.17 | | 67.49 | | 85.69 | | 58.01 | | 104.76 | | 46.04 | | 124.08 | | 32.08 | | 143.47 | | 16.61 | 75.02 | | | - |
|  |  | | 3 | | - | | 175.69 | | 20.89 | | 196.31 | | 42.28 | | 176.35 | | 63.95 | | 155.4 | | 85.81 | | 133.88 | | 107.79 | | 112.01 | | 129.86 | | 89.89 | | 151.99 | | 67.58 | | 174.17 | | 45.15 | | 196.4 | | 22.62 | 194.172 | | | - |
|  |  | | 5 | | - | | 218.5 | | 22.55 | | 208.54 | | 45.32 | | 186.07 | | 68.24 | | 163.28 | | 91.26 | | 140.27 | | 114.35 | | 117.11 | | 137.51 | | 93.83 | | 160.71 | | 70.47 | | 183.94 | | 47.03 | | 207.2 | | 23.54 | 214.64 | | | - |
|  |  | |  | |  | |  | |  | |  | |  | |  | |  | |  | |  | |  | |  | |  | |  | |  | |  | |  | |  | |  | |  | |  |  | | |  |
|  | 1 | | 1 | | - | | 186.43 | | 20.53 | | 194.09 | | 41.32 | | 173.47 | | 62.26 | | 152.37 | | 83.31 | | 130.98 | | 104.44 | | 109.4 | | 125.63 | | 87.69 | | 146.87 | | 65.87 | | 168.14 | | 43.97 | | 189.44 | | 22.01 | 183.35 | | | - |
|  |  | | 3 | | - | | 231.46 | | 23.37 | | 214.21 | | 46.82 | | 190.65 | | 70.33 | | 166.98 | | 93.88 | | 143.25 | | 117.46 | | 119.46 | | 141.07 | | 95.62 | | 164.69 | | 71.75 | | 188.33 | | 47.86 | | 211.98 | | 23.94 | 223.82 | | | - |
|  |  | | 5 | | - | | 238.66 | | 24 | | 218.44 | | 48.05 | | 194.31 | | 72.14 | | 170.12 | | 96.25 | | 145.89 | | 120.38 | | 121.63 | | 144.53 | | 97.34 | | 168.69 | | 73.02 | | 192.86 | | 48.7 | | 217.03 | | 24.35 | 233.71 | | | - |
|  |  | |  | |  | |  | |  | |  | |  | |  | |  | |  | |  | |  | |  | |  | |  | |  | |  | |  | |  | |  | |  | |  |  | | |  |

|  |  |  | **H1 = 0 H2 = 100** | | **H1 =10**  **H2 =90** | | **H1 =20**  **H2 = 80** | | **H1 = 30**  **H2 = 70** | | **H1 = 40**  **H2 = 60** | | **H1 = 50**  **H2 = 50** | | **H1 = 60**  **H2 = 40** | | **H1 = 70**  **H2 = 30** | | **H1 = 80**  **H2 = 20** | | **H1 = 90**  **H2 = 10** | | **H1 = 100**  **H2 = 0** | |
| --- | --- | --- | --- | --- | --- | --- | --- | --- | --- | --- | --- | --- | --- | --- | --- | --- | --- | --- | --- | --- | --- | --- | --- | --- |
| **K** | **φ** | **Q** | **I1** | **I2** | **I1** | **I2** | **I1** | **I2** | **I1** | **I2** | **I1** | **I2** | **I1** | **I2** | **I1** | **I2** | **I1** | **I2** | **I1** | **I2** | **I1** | **I2** | **I1** | **I2** |
|  |  |  |  |  |  |  |  |  |  |  |  |  |  |  |  |  |  |  |  |  |  |  |  |  |
| **500** | 0.1 | 1 | - | - | - | - | - | - | - | - | - | - | - | - | - | - | - | - | - | - | - | - | - | - |
|  |  | 3 | - | 8.91 | 1.21 | 13.63 | 2.61 | 12.83 | 4.2 | 11.88 | 6 | 10.77 | 8.04 | 9.49 | 10.32 | 8.02 | 12.86 | 6.35 | 15.68 | 4.47 | 18.14 | 2.29 | 7.25 | ***-*** |
|  |  | 5 | - | 15.96 | 2.73 | 27.61 | 6.05 | 26.79 | 10.03 | 25.54 | 14.75 | 23.82 | 20.28 | 21.57 | 26.7 | 18.71 | 34.07 | 15.19 | 42.47 | 10.94 | 49.84 | 5.68 | 17.35 | ***-*** |
|  |  |  |  |  |  |  |  |  |  |  |  |  |  |  |  |  |  |  |  |  |  |  |  |  |
|  | 0.5 | 1 | ***-*** | 89.05 | 22.59 | 198.76 | 50.99 | 201.61 | 83.75 | 195.53 | 119.45 | 181.38 | 156.92 | 160.53 | 195.36 | 134.44 | 234.26 | 104.42 | 273.32 | 71.5 | 312.36 | 36.5 | 202.07 | ***-*** |
|  |  | 3 | ***-*** | 391.97 | 43.27 | 403.82 | 87.22 | 361.29 | 131.54 | 317.54 | 176.1 | 273.05 | 220.79 | 228.09 | 265.59 | 182.82 | 310.44 | 137.32 | 355.35 | 91.66 | 400.29 | 45.88 | 402.54 | ***-*** |
|  |  | 5 | ***-*** | 449.86 | 45.99 | 423.22 | 92.27 | 377.1 | 138.74 | 330.56 | 185.32 | 283.73 | 231.99 | 236.7 | 278.71 | 189.52 | 325.48 | 142.25 | 372.28 | 94.89 | 419.11 | 47.47 | 438.26 | ***-*** |
|  |  |  |  |  |  |  |  |  |  |  |  |  |  |  |  |  |  |  |  |  |  |  |  |  |
|  | 1 | 1 | - | 397.65 | 42.15 | 396.23 | 84.59 | 353.28 | 127.2 | 309.76 | 169.92 | 265.91 | 212.72 | 221.86 | 255.58 | 177.66 | 298.49 | 133.35 | 341.44 | 88.96 | 384.41 | 44.51 | 376.7 | ***-*** |
|  |  | 3 | - | 467.96 | 47.15 | 431.14 | 94.38 | 383.48 | 141.67 | 335.72 | 188.99 | 287.87 | 236.35 | 239.98 | 283.74 | 192.04 | 331.14 | 144.07 | 378.56 | 96.06 | 425.99 | 48.04 | 451.59 | ***-*** |
|  |  | 5 | - | 480.42 | 48.26 | 438.53 | 96.56 | 389.95 | 144.9 | 341.31 | 193.26 | 292.62 | 241.65 | 243.9 | 290.05 | 195.16 | 338.46 | 146.39 | 386.88 | 97.61 | 435.3 | 48.81 | 469.87 | ***-*** |
